# Hypermutability of Damaged Single-Strand DNA Formed at Double-Strand Breaks and Uncapped Telomeres in Yeast *Saccharomyces cerevisiae*

**DOI:** 10.1371/journal.pgen.1000264

**Published:** 2008-11-21

**Authors:** Yong Yang, Joan Sterling, Francesca Storici, Michael A. Resnick, Dmitry A. Gordenin

**Affiliations:** Laboratory of Molecular Genetics, National Institute of Environmental Health Sciences, National Institutes of Health, Department of Health and Human Services, Research Triangle Park, North Carolina, United States of America; National Institute of Diabetes and Digestive and Kidney Diseases, United States of America

## Abstract

The major DNA repair pathways operate on damage in double-strand DNA because they use the intact strand as a template after damage removal. Therefore, lesions in transient single-strand stretches of chromosomal DNA are expected to be especially threatening to genome stability. To test this hypothesis, we designed systems in budding yeast that could generate many kilobases of persistent single-strand DNA next to double-strand breaks or uncapped telomeres. The systems allowed controlled restoration to the double-strand state after applying DNA damage. We found that lesions induced by UV-light and methyl methanesulfonate can be tolerated in long single-strand regions and are hypermutagenic. The hypermutability required PCNA monoubiquitination and was largely attributable to translesion synthesis by the error-prone DNA polymerase ζ. In support of multiple lesions in single-strand DNA being a source of hypermutability, analysis of the UV-induced mutants revealed strong strand-specific bias and unexpectedly high frequency of alleles with widely separated multiple mutations scattered over several kilobases. Hypermutability and multiple mutations associated with lesions in transient stretches of long single-strand DNA may be a source of carcinogenesis and provide selective advantage in adaptive evolution.

## Author Summary

A variety of error avoidance mechanisms assure low mutation rates across the genome. Genetic defects in DNA replication or repair can lead to genome-wide increase in mutation frequency that may result in cancer predisposition and genetic disease. Transient localized hypermutability drastically differs in its biological consequences from genome-wide mutators. Since genome-wide hypermutability can cause reduced fitness due to accumulation of dysfunctional alleles, mutators are under negative selection pressure. By contrast, there would be less selection against temporary hypermutability within limited genomic regions, suggesting a special role in adaptive evolution and carcinogenesis. Mechanisms of transient hypermutability are poorly understood. Long stretches of single-strand DNA have been implicated but not demonstrated as a source of localized transient hypermutability. Using sophisticated yeast genetic systems that we developed, we found that transient stretches of chromosomal single-strand DNA at double-strand breaks and that telomeres can tolerate multiple lesions and are highly prone to damage-induced mutations, including a very unusual class of widely spaced multiple mutations. The hypermutability relied on error prone translesion DNA synthesis. Our work demonstrates a simple in vivo mechanism for localized transient hypermutability extending over several kilobases that can result in widely spaced multiple mutations without severe mutation load in the rest of the genome.

## Introduction

Accurate DNA synthesis and repair of DNA lesions assure low rates of mutation. Genetic defects in either lead to increased, genome-wide mutator phenotypes which can manifest in cancer predisposition [1]. Strong mutators can cause reduced fitness due to the accumulation of dysfunctional alleles which place them under continual negative selection pressure [2],[3]. By contrast, there would be little selection against transient hypermutability within subpopulations of cells or within limited genomic regions [4]. High mutation frequencies during transient hypermutability might contribute to overall population mutability and could represent a prominent source of multiple mutations important for evolution and cancer. Transient increases in localized mutation can even be regulated and benefit an organism, as found for somatic hypermutability in the immunoglobulin genes where a coordinated system of proteins induce DNA damage and repair the lesions in an error prone manner [5].

Examples of unregulated transient hypermutability include multiple mutations [4],[6], adaptive mutations in non-dividing cells [7], meiosis-associated hypermutability [8], hypermutability associated with repair of double-strand breaks (DSBs) [9] and increased mutability associated with senescence in telomerase-deficient yeast [10]. While the mechanisms remain to be explained, transient stretches of single-strand (ss) DNA have often been proposed as intermediates in hypermutation. Extended regions of ssDNA would be expected in association with DSBs and with dysfunctional uncapped telomeres, where many kilobases of ssDNA can be formed by 5′→3′ resection [11],[12]. The genome would be expected to be especially vulnerable to lesions in ssDNA since repair mechanisms such as base-excision repair (BER), nucleotide excision repair (NER) and post-replication repair (PRR) operate only in double-strand (ds) DNA. Restoration of damaged ssDNA to a ds-state would be expected to require translesion DNA synthesis (TLS) polymerases that are tolerant of lesions in a template and are often error-prone [13] due to misincorporation during synthesis past lesions.

While error-prone restoration of damaged long stretches of ssDNA to dsDNA might explain many examples of transient hypermutability, this hypothesis has yet to be substantiated. Long tracts of ssDNA formed at DSBs and uncapped telomeres (and potentially in other cell contexts) are known to cause checkpoint responses, where the fate of the cell is determined by a balance of arrest, adaptation, recovery and repair processes [11],[14],[15]. Direct estimates of extent of damage tolerance and amounts of damage-induced mutations in ssDNA formed in a chromosomal context are required to establish that damage in long ssDNA actually leads to hypermutability rather than to cell death. We, therefore, designed systems in the budding yeast *Saccharomyces cerevisiae* for the generation of defined, persistent long stretches of transient ssDNA in the vicinity of a unique DSB or an uncapped chromosomal telomere. Lesions could be induced in the ssDNA before restoration to dsDNA. The consequences of restoration could be assessed with genetic reporters located within the regions that give rise to ssDNA. We demonstrate here that damage tolerance in stretches of long ssDNA formed at DSBs and uncapped telomeres results in extremely high frequencies of single and widely-separated multiple mutations caused by two major types of DNA damaging agents.

## Results

### DNA Damage Amplifies Hypermutability Associated with DSB Repair

**Figure 1:**
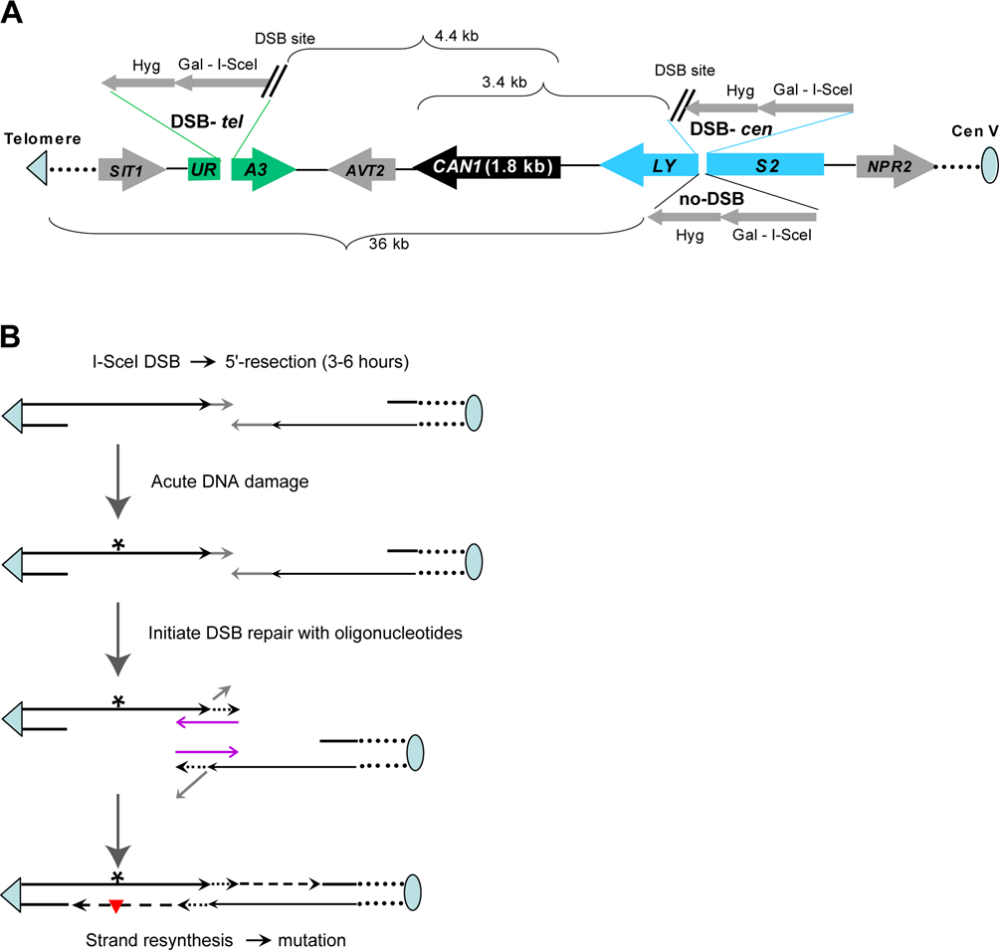
Mutagenesis in long ssDNA formed during repair of site-specific double-strand break. (A) Strains to assess DSB-repair-associated mutagenesis. *LYS2* and *URA3* genes were moved into positions near the forward mutation reporter *CAN1* in the left arm of chromosome V (shown not to scale; telomeric repeats are shown as a triangle). A self-generating DSB cassette [40] containing a hygromycin resistance gene, the I-*Sce*I cut site and the ORF for the I-*Sce*I endonuclease under the control of the inducible *GAL1*-promoter was placed in the middle of the *LYS2* (DSB-*cen*) or the *URA3* (DSB-*tel*) gene. The “no-DSB” control cassette in the *LYS2* location contained a non-cleavable I-*Sce*I half-site. (B) Damage-induced mutagenesis in long ssDNA formed at a site-specific DSB. The molecular mechanism of DSB-repair with oligonucleotide is shown as described for the “template” model in [16]. After 5′→3′ resection oligonucleotides anneal to the complementary parts of unresected strands on both sides of DSB. Non-complementary tails of unresected strands (grey lines) are removed and the new 3′-ends annealed to oligonucleotides are used to prime DNA synthesis (short-hatched lines with arrowheads). Second round of 5′→3′ resection removes oligonucleotides allowing repair via single-strand annealing between newly formed complementary ends of a DSB (not shown). Large ss-gaps created by resection should be restored to ds-state in order to accomplish repair. Acute DNA damage (indicated only in ssDNA by “*”) was applied after holding cells for 3 hr or 6 hr following DSB-induction to allow 5′→3′ resection.

Recently, we demonstrated that, following the induction of a DSB in a yeast chromosome by the I-*Sce*I site-specific endonuclease, there is 5′→3′ resection extending up to 20 kb from the break, after which repair can be mediated by a short oligonucleotide spanning the region of repair around DSB [16]. In this system, considerable DNA synthesis is required to restore the region to the ds-state. We employed this break-resection-oligonucleotide repair system to investigate the mutability of long ssDNA (Figure 1). Two types of strains were created wherein a cassette containing both an inducible Gal-I-*Sce*I and a DSB target was inserted into either *LYS2* on the centromere (DSB-*cen*) side or *URA3* on the telomere (DSB-*tel*) side of the forward mutation reporter *CAN1*. Placing cells in galactose medium for 3–6 hr led to induction of the DSB and resection. DNA damage was then induced by ultraviolet light (UV) or methyl methane sulfonate (MMS). The cells were subsequently transformed with a pair of 95 nt self-complementary oligonucleotides that also had sequence homology to both sides of the break to allow DSB repair. As described in [16] only a portion of the cells experience a DSB. The cells that repaired a DSB with oligonucleotides gave rise to colonies on selective medium lacking lysine (–Lys) or uracil (–Ura). No Lys^+^ or Ura^+^ colonies (<10^−9^) were observed in controls lacking oligonucleotides (data not shown).

In the absence of additional DNA damage the median frequencies of DSB-induced transformation in the wild-type varied from 48 to 205×10^−5^ for the cells with a break at the DSB-*cen* position and from 14 to 26×10^−5^ for a break at the DSB-*tel* position (Tables S1 and S2). While this was 10–100 fold lower than observed for an induced DSB at the *TRP5* gene on chromosome VII ([16] and Tables S1 and S2), the frequencies were 120–1300 fold in excess over the corresponding “no-DSB” controls (containing the Gal-I-*Sce*I insert, but lacking the I-*Sce*I cut site). DNA damage (UV or MMS) increased transformation frequencies in the no-DSB controls, possibly by introducing recombinagenic DNA lesions. However, cells experiencing a DSB followed by exposure to UV or MMS yielded transformation frequencies that were still considerably greater (40–160 times) than for the “no-DSB+DNA damage” controls. Thus, selection of individual DSB repair events resulting from restoration of a disrupted gene by oligonucleotides (Lys^+^ or Ura^+^ colonies) provides the opportunity to specifically examine subpopulations that had undergone DSB repair.

**Figure 2:**
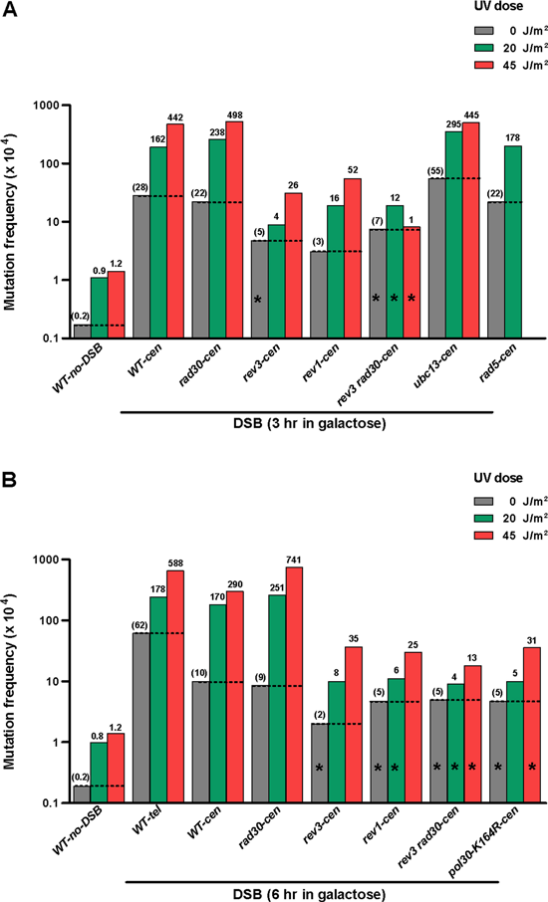
UV-mutagenesis associated with DSB-repair. (A, B) Wild-type and mutant yeast strains were incubated in galactose to induce a DSB for 3 hr (A) or 6 hr (B) and then treated with different doses of UV light. The *can1* mutant frequencies were determined among Lys^+^ (DSB-*cen*) or Ura^+^ (DSB-*tel*) transformants that arose after DSB induction and repair by oligonucleotides. Mutation frequencies determined in cell populations of strains containing the Hyg-Gal-I-*Sce*I insert lacking the I-*Sce*I cut site in the *cen* (*LYS2*) position served as “no-DSB” controls. The medians of mutation frequencies obtained in several independent transformation experiments are presented as log_10_ (mutation frequency×10^4^). The values of median frequencies, ranges of variation and numbers of repeats are provided in Tables S1 and S2. The heights of the bars represent either median frequencies, or the upper limit of 95% CI when the number of mutants was small (indicated with *). Numbers above bars represent frequencies of induced mutations for all variants involving DNA damage or frequencies of spontaneous mutations for “no damage” controls (shown in parentheses).

**Figure 3:**
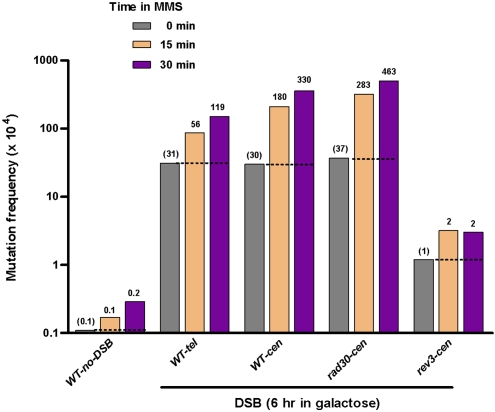
MMS-mutagenesis associated with DSB-repair in wild type (WT) and various mutants. Wild-type and mutant yeast strains were incubated in galactose to induce a DSB for 6 hr and then treated with 11.8 mM (0.1%) MMS (C) for 15 or 30 min. See Figure 2 legend for other details.

The frequencies of spontaneous *can1* mutants in the total populations of the DSB strains as well as in “no-DSB” control strains that were carried through all stages of transformation were 0.1–1.0×10^−4^ (Figures 2, 3 and Tables S1 and S2). However, the *can1* mutation frequencies were 50–300 times greater among the Lys^+^ (or Ura^+^) selected colonies that arose after DSB induction and oligonucleotide repair. This is in agreement with observations of increased spontaneous mutation rates in the vicinity of a DSB repaired by recombination with a homologous chromosome or with a repeat in the same chromosome [9],[17]. Doses of UV and MMS which caused 2–8 fold increases over low mutation frequencies in “no DSB” controls also amplified 3–30 times the high mutation frequencies associated with DSB repair (Figures 2, 3 and Tables S1 and S2). Importantly, the frequencies of damage-induced *can1* mutations among the cells that repaired a DSB were 180- to 1800-fold greater than in the population of the control, no-DSB strains treated with the same agents (Figures 2, 3). Strong increases in the frequencies of repair-associated *can1* mutants were observed regardless of whether the DSB was centromere or telomere proximal. The DSB-repair-associated spontaneous and damage-induced mutagenesis was observed only if the DSB was in the vicinity of the mutation reporter *CAN1*. A DSB in a different chromosome (*TRP5* in chromosome VII) did not affect mutagenesis in *CAN1* (Tables S1 and S2).

### Hypermutability Associated with Uncapped Telomeres

We utilized the special features of the *cdc13-1* mutation to explore damage-induced mutagenesis in ssDNA generated at the ends of chromosomes with uncapped telomeres. Cdc13, in complex with other proteins, protects chromosome ends after DNA replication by initiating telomere capping [12],[18]. The mutant *cdc13-1* is severely defective in postreplicative telomere capping at the nonpermissive temperature (37°C) resulting in G2/M arrest. This arrest is associated with up to 15 kb 5′→3′ end resection that can be detected by ssDNA formation in 3–10% of individual chromosome ends [18],[19].

**Figure 4:**
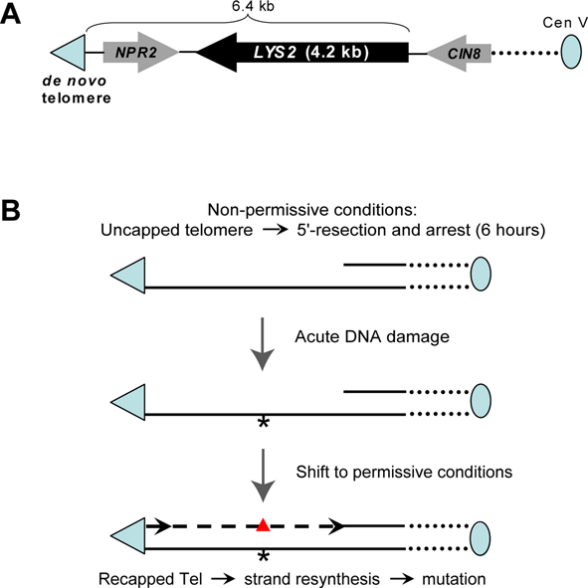
Mutagenesis in long ssDNA formed at uncapped telomere. (A) Strain to assess mutagenesis associated with an uncapped telomere. The 34 kb non-essential region between the original telomere and *NPR2* at the left end of the chromosome V was removed and the *LYS2* forward mutation reporter was inserted in the vicinity of the *de novo* telomere. (B) Damage-induced mutagenesis in long ssDNA formed at an uncapped telomere. Shifting of *cdc13-1* mutants to the non-permissive temperature (37°C) results in the formation of an uncapped telomere followed by G2 arrest and formation of ssDNA. After 6 hr incubation allowing 3′→5′ resection to occur, acute DNA damage (UV-C) was applied to cells (DNA damage is indicated only in ssDNA by “*”) and normal cycling was restored by returning cells to the permissive temperature (23°C).

**Figure 5:**
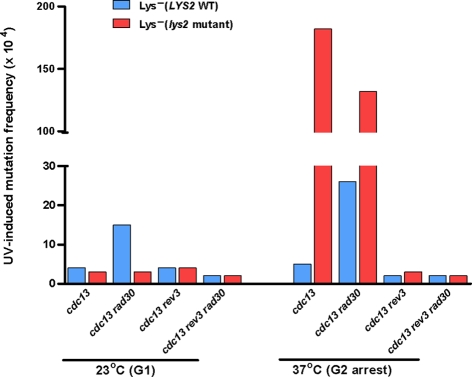
UV-induced mutagenesis associated with uncapped telomere arrest. Mutagenesis in the subtelomeric *LYS2* associated with uncapped telomere arrest. Frequencies of Lys^−^ mutants were measured in a *cdc13-1* strain without additional mutations (WT) or with additional mutations in TLS DNA polymerases. “23°C (G1)” - G1 saturated culture after incubation for 72 h at 23°C permissive temperature. “37°C (G2 arrest)”–G2 cells after additional incubation in fresh YPDA medium for 6 h at 37°C non-permissive temperature. Presented are frequencies of Lys^−^ mutants after UV irradiation (45 J/m^2^) since the numbers of spontaneous mutants were small. The frequencies and variation for spontaneous as well as UV-induced mutations are shown in Table S3. Frequencies are shown separately for the subtelomeric Lys^−^ mutants (Lys^−^ (*lys2* mutants)) and for Lys^−^ mutants containing mutations in genes other than *LYS2* (Lys^−^ (*LYS2* WT)).

In order to assess mutations in the subtelomeric region, 34 kb of nonessential DNA was removed from the left end of chromosome V and the mutation reporter *LYS2* was inserted near the *de novo* telomere (Figure 4A). The chromosome end was truncated to eliminate subtelomeric Y′ and X repeats in order to facilitate targeted integration and prevent silencing of the *LYS2* reporter [20]. After shifting to non-permissive temperature for 6 hr to cause G2/M arrest and 5′→3′ resection, the *cdc13-1* cells were UV-irradiated and plated onto rich (YPDA) medium. Mutagenesis was assessed by replicating colonies to –Lys medium. The frequency of *lys2* mutants among Lys^−^ colonies or sectors provided an estimate of mutagenesis next to a telomere. The appearance of Lys^−^ mutants in genes other than *LYS2* provided a comparison of telomere-associated *vs*. genome-wide mutagenesis within the same cell population. In addition to *LYS2*, there are at least 7 genes scattered across the genome mutations in which can lead to lysine auxotrophy ([21] and http://db.yeastgenome.org/cgi-bin/locus.pl?locuslys). Frequencies and allelism of Lys^−^ mutants were also determined in UV-irradiated cells that were not subjected to the 37°C arrest (Figure 5 and Table S3). Almost no mutants (<10^−4^) were observed in the no-UV controls. Exposure to 45 J/m^2^ had little impact on survival of the *cdc13-1* mutant regardless of irradiation at 37° or 23°C. However, the frequency of UV-induced *lys2* mutants in the 37°C-arrested cells was at least 60 times that in the non-arrested control cells kept at 23°C. The low frequency of genome-wide UV-mutagenesis, as indicated by Lys^−^ auxotrophic mutations at other loci (“Lys^−^ (*LYS2* WT)” bars), was not affected by the *cdc13-1* arrest at 37°C. Genome-wide location of mutations in the Lys- (*LYS2* WT)-category was confirmed by allelism test which assigned more than a half of them to 6 out of 7 genes with expected Lys- mutant phenotype (Table S3).

### UV-Induced Hypermutability Results in Widely-Spaced Multiple Mutations

PCR and sequencing analyses of spontaneous and UV-induced *can1* and *lys2* mutants did not identify any gross chromosome rearrangements which might occur at a low frequency through elimination of up to 43 kb of the nonessential region at the left end of the chromosome V [22]. The low frequency of GCR is likely due the high probability of loss of a chromosome having either an unrepaired DSB or an uncapped telomere. The following types of mutations were identified: substitutions of a single base pair (referred as simple base substitutions), small insertions or deletions of one to three adjacent nucleotide (indels) as well as complex mutations which included clusters of individual mutations separated by ≤10 nt (Tables S4, S5, S6, S7, S8, S9, S10, S11, and S12). With one exception all categories of mutations were found among the spontaneous and UV-induced mutants regardless of the possibility for ssDNA generation. These categories were also found in previous studies that examined spontaneous and UV-induced mutations in *can1* [23],[24].

The striking feature of UV-induced mutation spectra associated with DSB repair or with uncapped telomere arrest was the presence of many multiple mutant alleles with as many as 6 widely-spaced mutations (Table 1, Tables S4, S5, S6, S7, S8, S9, S10, S11, and S12; also see next section). The distances between adjacent mutations within multiple-mutant alleles were usually greater than 100 bp (∼90%), reaching more than a few thousand nucleotides in the longer *LYS2* gene. There was no apparent clustering of mutations (Tables S13 and S14) suggesting that they could have originated from independent UV-lesions within the same DNA molecule.

**Table 1:** Incidence of mutants with single and multiple mutations.

Target	Origin of mutants	Number of mutations in a mutant^c^						*D *(mut/kb)^d^
		1	2	3	4	5	6	**n.d.**
*CAN1*	no DSB; UV (45)^a^	43	1	0	0	0	0	n.d.
*CAN1*	DSB-cen; no UV	26	1	0	0	0	0	n.d.
*CAN1*	DSB-cen; UV (20)	17	6	2	0	0	0	0.53
*CAN1*	DSB-cen; UV (45)^b^	21	7	4	0	0	0	0.79
*CAN1*	DSB-tel; UV (45)	4	12	0	0	0	0	n.d.
Subtelomeric *LYS2*	23°C (no arrest); UV (45)	20	0	0	0	0	0	n.d.
Subtelomeric *LYS2*	37°C (arrest); UV (45)	7	16	5	4	1	1	0.34

a UV-doses (J/m^2^) are shown in parentheses.

b The category of *can1* mutants “DSB-*cen*, UV (45)” was combined from the group of single *can1* mutants (Table S7) and double *can1 ura3* mutants (Table S8) induced by 45 J/m^2^.

c The number of mutations in a mutant represents the total of all kinds of mutations identified in the mutant allele. Each complex mutation was counted as a single event.

d *D* (mut/kb)–densities of mutations calculated in assumption of Poisson distribution as described in Table S15 and Text S1.

The high incidence of multiple mutations indicates that the level of UV-induced mutagenesis associated with DSB-repair or uncapped telomere arrest is even greater than estimated based simply on frequencies of loss-of-function *CAN1* or *LYS2* mutations (Figures 2 and 5). Therefore, we sought to estimate the actual frequencies of mutation per kilobase based on truncated Poisson distribution as described in [25] (Table 1, Table S15 and Text S1). To estimate the probability of mutation it is necessary to know what fraction of all (single and multiple) mutant alleles result in loss of function. The fraction is expected to be smallest for single mutations and increase with multiplicity, thus multiple mutations would provide more accurate estimates of mutation probability. Therefore, for each set of data two types of calculations were developed based only on alleles with multiple mutations as well as based on the entire experimental distributions as described in Table S15. While both calculations gave comparable results, Table 1 presents calculations based on multiple mutations. Each truncated Poisson distribution was calculated with two assumptions: all alleles included in a calculation lead to loss of function and the probability of mutation is the same for all survivors. The range of calculated densities of UV-induced mutations was 0.8–2.2 per target (Table S15). In the case of hypermutability associated with uncapped telomere arrest we obtained a direct estimate of the mutation density. For that purpose we sequenced the1848 nt *NPR2* ORF located in the immediate vicinity of a telomere (Figure 4A). We looked for the presence *npr2* mutations in the subsets of UV-induced *lys2* mutants that originated from the G1 and G2-arrested *cdc13-1* cells. There were no *npr2* mutations associated with 9 *lys2* mutants originating from UV-irradiated G1-cells (Table S11); however, we found 8 mutations in the *npr2* ORFs of the 12 UV-induced *lys2* mutants associated with uncapped telomere arrest (P<0.02). The density of mutations in *NPR2* derived from this direct measurement is 0.36 mutations per kb, which is close to the density of mutations in the more distant telomere *LYS2* calculated on the basis of truncated Poisson distribution (Table 1). The fit between the experimental numbers of multiple mutants and the numbers expected from truncated Poisson distributions was high (0.4<P(_χ_^2^)<0.9) indicating that the mutations were independent and that there was no preferential loss of ssDNA molecules with higher density of damage within the population of damaged molecules. However, the relative survival of damaged *vs* undamaged ssDNA remains to be established using systems that would provide synchronous generation of ssDNA by 5′→3′ resection in all cells (such as site-specific DSB caused by inducible HO-endonuclease).

Extrapolations of the calculated mutation densities yielded probabilities of 0.55–0.88 that cells contain at least one mutation (P_mut_ = 1−P_0_). This is 10 to 100 times greater than the observed frequencies of UV-induced mutants associated with DSB repair or uncapped telomere arrest (Figures 2, 5 and Tables S1 and S3). This discrepancy could be explained if there is a high likelihood of UV-induced mutants not being detected and/or by a non-uniform distribution of UV-induced mutability among cells. The latter would be expected if UV-induced hypermutability in ssDNA is associated with variable amounts of 5′→3′ resection. In this case the extremely high likelihood of mutation would be a feature of only a small fraction of survivors where resected DNA extended through the reporter at the time of UV-irradiation. Regardless of the reason for the discrepancy, the high incidence of multiple mutant alleles provides evidence for exceptionally high levels of UV-induced hypermutability associated with DSB repair or uncapped telomere arrest.

### Strand Bias of UV-Induced Single and Multiple Mutations Identifies Stretches of ssDNA Associated with DSB-Repair and Uncapped Telomere Arrest as a Source of Hypermutability

**Figure 6:**
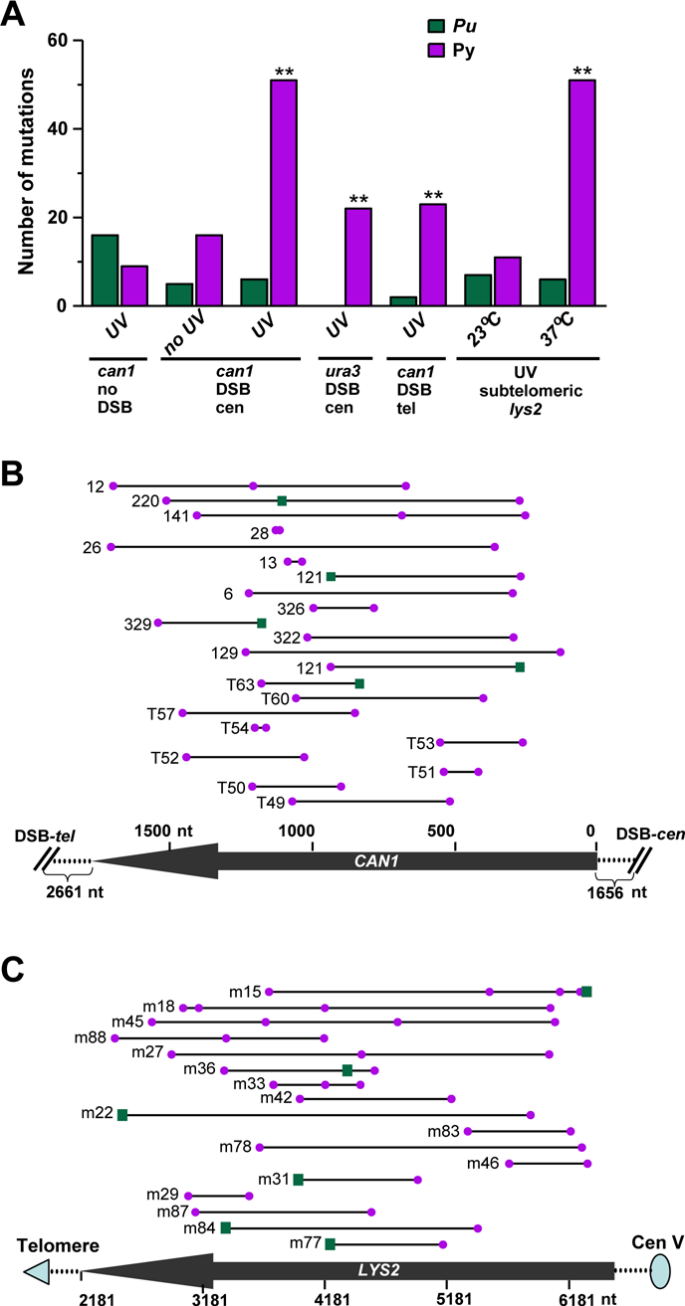
UV-induced and spontaneous mutants associated with DSB-repair and uncapped telomere arrest. (A) Nucleotide changes in simple base substitutions associated with single and multiple mutant alleles. Bars represent the numbers of simple base substitutions in purine (Pu) or pyrimidine (Py) nucleotides in the unresected strand or in the coding strand, if no resection was expected. Detailed information about DNA sequences and mutation types in all mutant alleles is provided in Tables S4, S5, S6, S7, S8, S9, S10, S11, and S12 and summarized in Tables S16, S17, and S18. All sequenced mutants were from the wild-type (for *can1* mutants) or *cdc13-1* (for *lys2* mutants) strains. All simple base substitutions from *can1* mutants induced by 20 and 45 J/m^2^ in the DSB-*cen* strains were pooled. Other categories are the same as in Table 1 and in Tables S16, S17, and S18. Statistically significant differences in comparison to the “no-DSB-UV” controls for *can1* mutants and to the “no arrest (23°C)” control for subtelomeric *lys2* mutants are indicated by “**”. (B, C) Simple base substitutions in multiple mutant alleles. Mutations of *CAN1* (B) are summarized from Tables S6, S7, and S8 and S10 and mutations of *LYS2* (C) from Table S12. Multiple mutant alleles are shown as lines connecting individual mutations. Substitutions in pyrimidines (purple balls) and substitutions in purines (green squares) are shown above their positions in either *CAN1* or *LYS2* ORF (bottom lines with the arrowheads). Base substitutions are shown for the unresected strand. Indels and complex mutations are not shown. For the subtelomeric *LYS2* reporter only distances from telomere are shown. Since presented *can1* mutant alleles were obtained in strains with a DSB-site placed either on telomeric (allele numbers with a prefix “T”) or on centromeric side (numbered without a prefix) of the *CAN1*, coordinates are given within the ORF. Also shown are the distances of the ORF ends from a break in each type of constructs (DSB-*tel* or DSB-*cen*).

The primary products of UV-damage as well as sources of mutations are cyclobutane pyrimidine dimers and 6-4 photoproducts resulting from covalent linkages of adjacent pyrimidines [26]. Nearly all UV-induced simple base substitutions (substitutions that were not the part of a complex mutation) in single and multiple mutants associated with DSB-repair (*can1*) or uncapped telomere arrest (*lys2*) could be attributed to pyrimidine bases of the unresected strand (Figure 6A and Table S16). Importantly, the bias was always directed to changes at pyrimidines of the unresected strand regardless of the position of the DSB (compare DSB-*cen* and DSB-*tel*). The large majority of pyrimidines mutated in the unresected strand were associated with di-pyrimidines (128 out of 147; derived from data in Table S16). A strong bias towards pyrimidines in unresected strands was also observed for base substitutions identified within UV-induced complex mutations associated with DSB-repair and with uncapped telomere arrest (33 pyrimidines∶1 purine; derived from data in Table S18). The strand bias associated with ssDNA-forming conditions contrasted with the nearly equal frequencies of UV-induced substitutions of purines and pyrimidines in either strand in the absence of a DSB or *cdc13-1* arrest (Figure 6A and Table S16). Additional strand bias analysis for 39 multiple mutant alleles of *CAN1* and *LYS2* with more than one simple base substitution (Figures 6B and 6C) revealed that only 11 of 92 base substitutions in these alleles could be assigned to purines in the unresected strand (P<0.001 for the hypothesis that mutations in purines and pyrimidines occur with probabilities proportional to their presence in the *CAN1* and *LYS2* ORFs). This supports the view that multiple mutations resulted mostly from independent UV-photoproducts rather than from extended, error-prone DNA synthesis triggered by a single UV lesion.

In the DSB-*cen* strains the *URA3* gene was located telomere proximal to *CAN1* (Figure 1A). In a small fraction (1–3%) of UV-induced *can1* mutants associated with DSB-repair the *URA3* gene was also inactivated, mostly by single mutations (Table S9). Although the number was small, multiple mutations were also observed in *URA3*. The lower incidence of multiple mutations could be due to the size of the ORF (804 nt, *URA3*; 1773 nt, *CAN1*), a potentially lower probability of *URA3* inactivation by mutations as well as to more distant location from a DSB than *CAN1*. Importantly, all 22 base substitutions identified in *URA3* resulted from mutations in pyrimidines in the strand that would remain after 5′→3′ resection into this gene. In total *can1 ura3* double mutants (Tables S8 and S9) contained from two to four mutations (*e.g.*, isolates #15 and #28) most of which were strand-specific. Based on reported lengths for 5′→3′ resection at a site-specific DSB ([11] and references therein), we propose that the multiple UV-induced mutations in both *URA3* and *CAN1* originated from damage in large continuous stretches of ssDNA. For the case of the induced DSB, the continuous ssDNA regions would have to be >6.3 kb (distance between the DSB-*cen* and the 5′-end of *URA3*; see Figure 1A) and >6.4 kb for the uncapped telomere (distance between the Tel-*de novo* and the 5′-end of *LYS2*; see Figure 4A). The continuity of subtelomeric hypermutable regions can also be derived from the observed multiple, strand-biased UV-induced mutations in *NPR2* and *LYS2* (previous section and Table S12). These observations strongly support the view that the hypermutable region is created by 5′→3′ resection starting at the telomere and extending through both ORFs. Altogether, the strong strand bias of UV-induced single and multiple mutations demonstrates that long stretches of damaged ssDNA are the source of mutagenesis and that the ssDNA can be successfully restored to the ds-state even if it contains multiple lesions.

### Role of Translesion Synthesis in Hypermutability Associated with ssDNA

To address the mechanism(s) of mutation during restoration of damaged ssDNA to complete chromosomes, we investigated the roles of genes involved in translesion DNA synthesis (TLS) and post-replication repair (PRR) on mutagenesis associated with DSB-repair (Figures 2, 3 and Tables S1 and S2).

Similar to the other forms of damage-induced mutagenesis [13], hypermutability associated with DSB-repair required the translesion polymerase (Pol ζ. Deletion of *REV3*, encoding the catalytic subunit of Pol ζ eliminated most of the UV- and MMS-induced mutagenesis while removal of the translesion polymerase Pol ζ (*rad30Δ*) had no detectable effect (Figures 2 and 3). The remaining level of UV-mutagenesis associated with DSB-repair was generally decreased further in a *rev3Δrad30Δ* double mutant. We also examined the impact of a *rev1Δ* mutation [27] and a PCNA-mono-ubiquitination defect (*pol30-K164R*) [28]), which can lead to indirect disruption of Pol ζ TLS function. Both defects also prevented the DSB repair-associated mutagenesis providing additional support that mutagenesis relies on Pol ζ. Mutagenesis was not affected by deletions of the *UBC13* or *RAD5* genes, responsible for steps in PCNA poly-ubiquitination and error-free damage avoidance by template switching [28]. Similar to findings by Jeff Strathern and co-authors [17], spontaneous hypermutability associated with DSB-repair was also reduced 2–30 fold by inactivation of Pol ζ mediated translesion synthesis (Figures 2 and 3). Possibly there is damage to ssDNA prior to DSB repair that results in spontaneous hypermutability.

We considered the possible contribution of greater variability and apparent overall reduction in transformation frequencies, regardless of DNA damage, to these results. For example, if transformants arose through recombination between unbroken chromosomal DNA and oligonucleotides [29], then there would be little contribution of ssDNA to mutagenesis. However, we found that most transformants were indeed associated with DSB repair. In experiments involving MMS treatment of the various mutants, the DSB-induced transformation frequencies were 30- to 1000-fold higher than found with the wild type no-DSB controls (Table S2). For the case of UV-irradiation, some TLS polymerase single and double mutants showed as little as a 2- to 10-fold increase in transformation frequencies over wild type no-DSB controls, while others had >10-fold increases (Table S1). However, since even for the 2-fold increase at least 50% of the transformants could be assigned to events associated with oligonucleotide mediated DSB repair, drastic effects of TLS mutations on reductions in mutagenesis could be detected.

Similar to the observations with DSB-repair associated mutagenesis, the UV-induced hypermutability of *LYS2* associated with uncapped telomere arrest depended on the translesion Pol ζ (*REV3*) and was not affected by elimination of Pol ζ (*RAD30*) (Figure 5). (Note: the *rad30* mutation caused a moderate increase in the frequency of Lys^−^ (*LYS2* ^+^) cells independently of uncapped telomere arrest. This could be due to a stronger role for Pol η in error-free TLS among the unidentified *LYS* genes as compared to *LYS2*.)

## Discussion

Utilizing systems that can create resected chromosomal DNA, we found that long stretches of ssDNA formed at DSBs and uncapped telomeres (>6 kb based on the region of detectable mutations) can be efficiently restored to the ds-state in spite of the presence of multiple UV-induced lesions. Lesions in ssDNA along with ssDNA itself were hypermutable and hypermutability relied on TLS Pol ζ. We established the involvement of a ssDNA intermediate in transient hypermutability based on the strand-biased spectrum of UV-induced single and multiple mutations. Importantly, strand biased multiple mutations provide a useful tool for detecting in vivo mutagenesis in single molecules containing long stretches of damaged ssDNA. UV or any other mutagen with a bias towards certain DNA bases would create a permanent signature of strand-biased multiple mutations in chromosomal regions containing long stretches of ssDNA at the time of acute induction of lesions. Importantly, the density of UV-induced mutations was estimated to be ∼1 per 1.5–3 kb, which is comparable to the density of pyrimidines dimers induced by UV as determined in yeast [30],[31]. Thus, we conclude that many UV-lesions in ssDNA directly result in mutations during the DNA synthesis that leads to chromosome restoration. Based on the similarity in genetic controls and responses to UV and MMS, we propose that there is a general phenomenon whereby lesions in long ssDNA tracts lead to hypermutagenesis. Remarkably, the mutation frequencies can reach 0.4–0.8 per kb (Table 1). This is comparable to the very high frequencies found for somatic hypermutation of immunoglobulin genes [5]. Since survival in our experiments is high and the mutation load in the rest of the genome is low, we anticipate that damage-induced localized hypermutability associated with ssDNA can achieve extremely high levels.

Transient long stretches of ssDNA can form in several ways. Tens of kilobases of ssDNA can be formed in yeast by 5′→3′ resection at site-specific DSBs [11] and uncapped telomeres [12]. Recently, we found that ssDNA is also formed by resection at random DSBs induced by ionizing radiation (J. Westmoreland, W. Ma and M. Resnick, unpublished). Also, there are strong indications of ssDNA formation at DSBs in mammalian cells [32],[33]. Since delays in DSB-repair or in telomere recapping would increase the time of exposure and the length of ssDNA tract, resulting in hypermutability, it will be important to identify factors responsible for coordination of steps in DSB-repair and/or telomere maintenance. These factors would suppress the potential for hypermutability associated with ssDNA.

In addition to end-resection, there are several possible sources of ssDNA. Long ssDNA has been detected in cultured human lymphoma cells, although the mechanism of generation has not been ascertained [34]. Uncoupled leading and lagging strand synthesis resulting from occasional disruption of the replisome or DNA damage can lead to ssDNA regions [35],[36]. The ssDNA created by replication fork uncoupling might be highly prone to damage-induced mutagenesis. The similarity in hypermutability associated with DSB-repair and uncapped telomere suggests that there is generally a strong potential for genome instability associated with transient stretches of long ssDNA.

We observed a high incidence of widely-spaced multiple mutations caused by damage in ssDNA. Multiple mutations are an important genetic phenomenon that may affect species evolution and cancer incidence [4],[6],[37]. They have a greater likelihood of conferring a selective advantage as compared with each single mutation which might have just a modest, neutral or even negative effect on gene function [38]. Our work provides a simple molecular mechanism for the simultaneous occurrence of multiple mutations across regions that may be several kilobases long, recently described as “mutation showers” [6].

## Material and Methods

### Strain Construction

All yeast strains were isogenic to CG379 [39] with the following common markers, *MATα ade5-1 his7-2 leu2-3*,*112 trp1-289 ura3*Δ All strain constructions were performed using methods of PCR-based gene disruption and direct genome modification by oligonucleotides as described in [16],[40],[41] and references therein. Deletion strains for genetic control studies were generated by inserting antibiotic resistance markers as described [42]. Single deletions were obtained by inserting the G418 resistance *(kanMX4)* module in place of the chosen ORF. Double-deletion strains were obtained by switching the G418 resistance marker to nourseothricin resistance (*kanMX4* to *natMX4*) followed by *kanMX4* insertion to replace the second ORF. All construction steps were verified by phenotype and by PCR. The site-specific *pol30-K164R* mutant was generated as described in [43] and verified by sequencing. The strain FRO-1 with a self-generating DSB cassette inside the *TRP5* gene in the chromosome VII used in control experiments has been described [16].

### Yeast Strains for Assessing Mutagenesis Associated with Double-Strand Break Repair (Figure 1A)

At the first step of construction the *LYS2* gene was inserted after position 34,193 nt in the chromosome V with the help of the tailed PCR primers: oYY_a - TTCTTACTCAGTGTGAACGTGTTCTAAATAAGTTCTTGTTCTAATTAATT-**TAAGCTGCTGCGGAGCTTCC** and oYY_b - AGATACGATTACTCCAGTTCCTCTTACAAGAAATGCATAAAAATAGTTAC-**AATTACATAAAAAATTCCGGCGG** (targeting tails are underlined; *LYS2*-amplification tails are in shown in bold). Then, a CORE-I-*Sce*I cassette containing the I-*Sce*I gene under control of the *GAL1* promoter, the hygromycin resistance gene (*Hyg*) and an I-*Sce*I site was placed inside the *LYS2* flanked by the following sequences: GCTTGCCGACGGCGGCTAAGCTCATAACATTGATAGTTGAAATAACATTTGGA - telomere proximal and CACAGTTGATATAATTATCCATAATGGTGCGTTAGTTCACTGGGTTTATCCATATGCCAAATTGAGGGA–centromere proximal (insertion strategy and sequences of oligonucleotides are available upon request). At the second step the *URA3* gene was inserted after position 29,682 in chromosome V with the help of the tailed PCR primers: oYY_5 - CAGTCTTTATATCAGTCTTTGCATGGCTTTGCATCTGATGCTGGCTCTACCGACTTCTCG-**CAGAGCAGATTGTACTGAGAGTGCACC** and oYY_6 - ACTCAAATATTTATCCACTTTGGATAGTATATACGTCAAATTCTTTTGGTATTTTATCGC-**CGCATCTGTGCGGTATTTCACACCGC** (targeting tails are underlined; *URA3*-amplification tails are in shown in bold). Two independent isolates, YY-22 and YY-24 were used as wild type DSB-*cen* strains for this study. The control “no DSB” strains (YY_122 and YY_124) differ from YY_22 and YY_24 in that they contain uncuttable, incomplete (half) I-*Sce*I sites.

In order to place the self-generating DSB cassette on the telomere side of *CAN1* (DSB-*tel*) we first transformed YY_22 and YY_24 strains to Lys+ with “repairing” oligonucleotides oYY_15 and oYY_16 (see below) and selected the removal of the Gal-I-*Sce*I-CORE insert from the *LYS2* gene. Then, a CORE-I-*Sce*I cassette containing the I-*Sce*I gene under the *GAL1* promoter, hygromycin resistance gene (*Hyg*) and an I-*Sce*I site was placed inside the *URA3* gene flanked by the following sequences: CAAGGAATTACTGGAGTTAGTTGAAGCATTAGGT (telomere proximal) and TATCCACATGTGTTTTTAGTAAACAAATTTTGGG (centromere proximal) to produce two identical DSB-*tel* strains YY-266 and YY-267.

### Yeast Strains for Assessing Mutagenesis Associated with Uncapped Telomere Arrest (Figure 4)

In order to generate a chromosome end-truncation and also create a *de novo* telomere we inserted Gal-I-*Sce*I CORE after 34 193 nt at the left end of chromosome V (sequence context: TTCTTACTCAGTGTGAACGTGTTCTAAATAAGTTCTTGTTCTAATTAATT–on the telomere side and GTAACTATTTTTATGCATTTCTTGTAAGAGGAACTGGAGTAATCGTATCT–on the centromere side). After induction of the DSB, cells were transformed with the oligonucleotide **TGTGTGTGGGTGTGGTGTGTGTGTGGGTGTGGTG**-GTAACTATTTTTATGCATTTCTTGTAAGAGGAACTGGAGTAATCGTATCT consisting of a short stretch of T(G)_1–3_ telomeric repeats (shown in bold) followed by sequence homologous to the DSB-end on the centromere side. Loss of CORE was selected and isolates with end-truncation and *de novo* telomere V were identified by both Southern hybridization of the *Pst*I-digested genomic DNA with *NPR2*-probe and by PCR with the following pair of primers: CA-16–CACCACACCCACACAC, telomere repeat-specific and Tel5-FS-npr2–GAACATTTTGCCCAGCCTAGTA, NPR2-specific. Based on these tests the size of added telomeric repeats was 300–500 nt (not shown). The *LYS2* ORF with promoter was amplified with targeting tailed primers oYY_9 (TAATATTACAACTTATTTCCGTAAATAAAGATAGTACACACGAATCCAAACGTTTATATAGTTAGCTCTG-TAAGCTGCTGCGGAGCTTCC ) and oYY_10 (AGACAGAAGAGAAGGGTGTGAAACCACCTCTACCAAACACACCAAGAGATGAACCTAAATCAAATTTTCA-AATTACATAAAAAATTCCGGCGG ) and inserted between *NPR2* and *CIN8* close to the *de novo* telomere (Figure 4A). Strain DAG_760 was generated by introducing the *cdc13-1* temperature-sensitive mutation into this strain with the help of the replacement plasmid pVL2862 containing the mutant *cdc13-1* allele and the *URA3* marker (gift from Vickie Lundblad). *cdc13-1* strains were always grown at permissive temperature 23°C. Deletion mutants in the genes *rev3* and *rad30* controlling TLS polymerases were obtained in the strain DAG_760. Two independent deletion isolates was studied for each genotype.

### Mutagenesis Associated with DSB-Repair

Yeast strains were grown with agitation in liquid rich media (YPDA) for approximately 20 hr and then diluted 37 fold with fresh 2% galactose synthetic complete media to induce Gal-I-*Sce*I expression for generation of a site-specific DSB. After 3 or 6 hr of growth in galactose cells were washed once with 50 ml dH_2_O and DNA damage was applied to the yeast suspension. The “No-DSB” control strains went through the same incubation and transformation steps as the strains that experienced an inducible DSB. In the case of UV, yeast were suspended in 25 ml of dH_2_O (∼10^6^ cells/ml), placed into a 150 mm Petri dish and irradiated with UV-C (254nm, 1 J/m^2^×sec) with continuous agitation for 20 or 45 seconds. In the case of MMS, cells were suspended in 5 ml of 50 mM sodium phosphate buffer (pH 7.0) (∼10^7^ cells/ml) and then treated with 11.8 mM (0.1%) MMS for 15 or 30 min. MMS was inactivated by adding Na_2_S_2_O_3_ to a final concentration of 5% (w/v) for 2 minutes. Treated and untreated yeast were washed once with 50 ml water followed by transformation to Lys^+^ or Ura^+^ in DSB-*cen* or DSB-*tel* strains, respectively, with a pair of complementary oligonucleotides (Figure 1) introducing a silent change (shown in small letters) creating additional restriction sites (underlined), *Ava*II (DSB-*cen*) or *Apa*I (DSB-*tel*). DSB-*cen* strains were transformed with a pair of oligonucleotides oYY_15–AATGGTGCGTTAGTTCACTGGGTTTATCCATATGCCAAATTGAGGGAcCCAAATGTTATTTCAACTATCAATGTTATGAGCTTAGCCGCCGTCGG and oYY_16 - CCGACGGCGGCTAAGCTCATAACATTGATAGTTGAAATAACATTTGGgTCCCTCAATTTGGCATATGGATAAACCCAGTGAACTAACGCACCATT; DSB-*tel* were transformed with a pair of oligonucleotides oYY_47–TGTTCGTACCACCAAGGAATTACTGGAGTTAGTTGAAGCATTAGGgCCCAAAATTTGTTTACTAAAAACACATGTGGATATCTTGACTGATTTTT and oYY_48 - AAAAATCAGTCAAGATATCCACATGTGTTTTTAGTAAACAAATTTTGGGcCCTAATGCTTCAACTAACTCCAGTAATTCCTTGGTGGTACGAACA. *can1* mutants were screened among Lys^+^ or Ura^+^ transformant colonies after replica plating onto media with 60 mg/ml of L-canavanine.

### Mutagenesis Associated with Uncapped Telomere Arrest

The *cdc13-1* strain DAG_760 or its derivatives with additional mutations in TLS polymerases, carrying *LYS2* next to the *de novo* telomere in the truncated left arm of the chromosome V (Figure 4A), were grown with agitation for 72 hr in YPDA at the permissive temperature 23°C. At this point the cell density reached 1 to 2×10^8^ cells/ml and more than 95% cells were in G1, based on cell morphology. Part of the culture was left at 23°C while the other part was diluted ten times with fresh YPDA and incubated at the non-permissive temperature 37°C for 6 hr. After additional incubation all cultures were diluted to 1–5×10^5^ cells/ml. A portion of the suspensions were UV-irradiated (45 J/m^2^). All suspensions were plated onto YPDA to yield around 100–500 colonies per plate. Colonies were replica plated onto synthetic complete and lysine deficient media. Single cell isolates from Lys^−^ colonies or sectors were then verified for the phenotype and checked for allelism to the *LYS2* gene.

## Supporting Information

Table S1 Frequencies of spontaneous and UV-induced *can1* mutants associated with DSB-repair.
(0.03 MB PDF)

Table S2 Frequencies of spontaneous and MMS-induced *can1* mutants associated with DSB-repair.
(0.02 MB PDF)

Table S3 Frequencies of UV-induced Lys^−^ mutants associated with uncapped telomere arrest.
(0.02 MB PDF)

Table S4 Mutation spectrum in the category “*can1* - no DSB; UV, 45 J/m^2^”.
(0.02 MB PDF)

Table S5 Mutation spectrum in the category “*can1* - DSB-cen; no UV”.
(0.02 MB PDF)

Table S6 Mutation spectrum in the category “*can1* - DSB-cen; UV, 20 J/m^2^”.
(0.02 MB PDF)

Table S7 Mutation spectrum in the category “*can1* - DSB-*cen*; UV 45 J/m^2^”.
(0.02 MB PDF)

Table S8 Mutation spectrum in the category “*can1* (from *can1 ura3* set) - DSB-*cen*; UV, 45 J/m^2^”.
(0.02 MB PDF)

Table S9 Mutation spectrum in the category “*ura3* (from *can1 ura3* set) - DSB-*cen*; UV, 45 J/m^2^”.
(0.02 MB PDF)

Table S10 Mutation spectrum in the category “*can1* - DSB-*tel*; UV, 45 J/m^2^”.
(0.02 MB PDF)

Table S11 Mutation spectra in the category “subtelomeric *lys2*, 23°C (no arrest) UV, 45 J/m^2^”.
(0.04 MB PDF)

Table S12 Mutation spectrum in the category “subtelomeric *lys2*, 37°C (arrest) UV, 45 J/m^2^”.
(0.04 MB PDF)

Table S13 Separation between mutations within double and triple mutant *can1* alleles.
(0.03 MB PDF)

Table S14 Separation between mutations within double and triple mutant *lys2* alleles.
(0.03 MB PDF)

Table S15 Calculated densities of UV-induced mutations.
(0.02 MB PDF)

Table S16 Simple base sustitutions.
(0.03 MB PDF)

Table S17 Simple indels.
(0.03 MB PDF)

Table S18 Base substitutions within complex mutations.
(0.03 MB PDF)

Text S1 Supplemental calculations.
(0.02 MB PDF)
