# Supplementary material for: Hypermutability of Damaged Single-Strand DNA Formed at Double-Strand Breaks and Uncapped Telomeres in Yeast Saccharomyces cerevisiae
Source: PLoS Genet. 2008 Nov 21;4(11):e1000264. doi: 10.1371/journal.pgen.1000264 (PMC2577886; doi:10.1371/journal.pgen.1000264)
Supplement: Table S1 — Frequencies of spontaneous and UV-induced can1 mutants associated with DSB-repair. (0.03 MB PDF) [file pgen.1000264.s001.pdf]

Table S1. Frequencies of spontaneous and UV-induced *can1* mutants associated with DSB-repair

DSB position [1]	Genotype	Time in galactose, hr	UV dose, J/m^2	Frequency of *can1* mutants (x 10^4)				Transformation to Lys^+ or Ura^+		% UV-survival (median) [5]
				among transformants		in population				
				median [2]	range [# of repeats] [3]	median	range	median frequency (x 10^7)	Fold over "no-DSB" control	
no	Wild type	3	0	NA	NA	0.17	(0.16-0.29)	16	NA	100
no	Wild type	3	20	NA	NA	1.1	(1.0-1.4)	135	NA	90
no	Wild type	3	45	NA	NA	1.4	(1.3-1.6)	230	NA	80
no	Wild type	6	0	NA	NA	0.19	(0.12-0.54)	47	NA	100
no	Wild type	6	20	NA	NA	0.99	(0.65-1.1)	180	NA	90
no	Wild type	6	45	NA	NA	1.4	(1.0-1.8)	130	NA	80
tel	Wild type	6	0	62	(19-81) [8]	0.10	(0.090-0.22) [4] [4]	2,600	NA	100
tel	Wild type	6	20	240	(120-370) [4]	1.0	(0.80-1.3)	1,750	NA	70
tel	Wild type	6	45	650	(330-950) [4]	3.1	(2.3-3.6)	1,550	NA	30
cen	Wild type	3	0	28	(20-33) [6]	1.0	(0.68-1.3)	20,500	1,281	100
cen	Wild type	3	20	190	(110-280) [8]	1.9	(1.2-4.5)	19,027	141	80
cen	Wild type	3	45	470	(400-630) [8]	3.6	(1.8-6.8)	10,283	45	60
cen	Wild type	6	0	9.9	(6.3-15) [12]	1.0	(0.85-1.2)	5,625	120	100
cen	Wild type	6	20	180	(140-320) [10]	1.8	(1.1-3.4)	7,235	40	80
cen	Wild type	6	45	300	(220-610) [10]	2.8	(2.1-4.4)	6,532	50	40
cen	*rad30*	3	0	22	(14-29) [4]	1.0	(0.94-1.5)	11,795	737	100
cen	*rad30*	3	20	260	(200-340) [4]	1.7	(1.6-1.8)	8,615	64	80
cen	*rad30*	3	45	520	(420-570) [4]	3.3	(3.0-4.9)	7,764	34	40
cen	*rad30*	6	0	8.6	(2.6-13) [8]	1.3	(1.1-1.8)	1,600	34	100
cen	*rad30*	6	20	260	(190-440) [6]	4.9	(3.9-6.3)	1,599	9	50
cen	*rad30*	6	45	750	(560-900) [6]	2.9	(2.8-3.1)	487	4	30
cen	*rev1*	3	0	3.1	(2.7-3.6) [4]	1.2	(1.0-1.3)	1,162	73	100
cen	*rev1*	3	20	19	(13-31) [4]	0.7	(0.49-0.82)	1,014	8	40
cen	*rev1*	3	45	55	(45-63) [4]	0.4	(0.38-0.47)	525	2	30
cen	*rev1*	6	0	<4.7 {6}	[6]	0.8	(0.61-0.83)	1,087	23	100
cen	*rev1*	6	20	<11 {7}	[6]	0.5	(0.33-0.59)	796	4	40
cen	*rev1*	6	45	30	(16-38) [4]	0.6	(0.51-0.61)	771	6	30
cen	*rev3*	3	0	<4.7 {2}	[6]	0.82	(0.30-1.1)	875	55	100
cen	*rev3*	3	20	8.9	(7.8-21) [6]	0.26	(0.18-0.34)	1,051	8	40
cen	*rev3*	3	45	31	(26-45) [4]	0.53	(0.38-0.68)	515	2	30
cen	*rev3*	6	0	<2.0 {4}	[6]	0.81	(0.69-1.90)	888	19	100
cen	*rev3*	6	20	9.8	(6.2-21) [6]	0.50	(0.33-0.97)	465	3	50
cen	*rev3*	6	45	37	(25-52) [4]	0.59	(0.50-0.63)	465	4	40

Table S1. (continued)

DSB position[1]	Genotype	Time in galactose, hr	UV dose, J/m²	Frequency of *can1* mutants (x 10⁴)				Transformation to Lys⁺ or Ura⁺		% UV-survival (median)[5]
				among transformants		in population				
				median [2]	range [# of repeats] [3]	median	range	median frequency (x 10⁷)	Fold over "no-DSB" control	
cen	*rad30, rev3*	3	0	<7.4 {2}	[6]	0.79	(0.48-0.83)	948	59	100
cen	*rad30, rev3*	3	20	<19 {4}	[6]	0.34	(0.24-1.1)	1,618	12	30
cen	*rad30, rev3*	3	45	<8.2 {0}	[6]	0.25	(0.22-0.36)	632	3	20
cen	*rad30, rev3*	6	0	<5.3 {1}	[4]	0.86	(0.73-0.96)	662	14	100
cen	*rad30, rev3*	6	20	<9.0 {0}	[4]	0.33	(0.25-0.44)	550	3	30
cen	*rad30, rev3*	6	45	<18 {0}	[4]	0.47	(0.33-0.65)	558	4	10
cen	*pol30-K164R*	6	0	<4.7 {12}	[8]	0.70	(0.43-1.2)	5,699	121	100
cen	*pol30-K164R*	6	20	10	(4.0-17) [12]	0.24	(0.21-0.60)	8,042	45	16
cen	*pol30-K164R*	6	45	<36 {3}	[4]	0.22	(0.19-0.21)	1,162	9	10
cen	*rad5*	3	0	22	(20-41) [6]	1.7	(1.4-1.9)	2,193	137	100
cen	*rad5*	3	20	200	(86-250) [6]	1.6	(0.81-1.9)	3,643	27	40
cen	*ubc13*	3	0	55	(45-130) [6]	1.2	(0.87-1.5)	2,751	172	100
cen	*ubc13*	3	20	350	(310-440) [6]	2.2	(1.4-3.0)	5,660	42	80
cen	*ubc13*	3	45	500	(470-690) [4]	2.5	(2.2-3.2)	2,165	9	40
TRP5 (Chr. VII)	Wild type	6	0	0.32	(16-0.65) [4]	0.17	(0.13-0.17)	195,000		100
TRP5 (Chr. VII)	Wild type	6	20	1.9	(1.3-2.6) [4]	1.3	(1.1-1.3)	34,500		30

[1] The "no-DSB" cassette was placed into the *LYS2* gene at the same position as the DSB-*cen* cassette (Figure 1A).

[2] Low frequencies were estimated using total number of mutants in all experimental repeats assuming Poisson distribution; total number of mutants obtained in all experiments with small numbers is shown in { }.

[3] There were 4 repeats for the "no-DSB" control.

[4] Frequencies of *can1* mutants in the population was calculated in 4 out of 8 repeats for this variant.

[5] UV-survival was estimated in the population of cells plated after transformation procedure. The procedure included resuspending and centrifugation steps in water as well as in viscous PEG solution, which could lead to uncontrolled loss of cells. Therefore, the numbers were rounded to the first significant digit. The UV-survivals for different genotypes were comparable to the reported values (McDonald et al., 1997; Stelter and Ulrich, 2003; Torres-Ramos et al., 2002) and to the UV-survival for the *rev3*, *rad30* and *rev3 rad30* mutants in the experiments with *cdc13-1* background, in which there was no transformation procedure (Table S3).

References to Table S1

McDonald, J.P., Levine, A.S., and Woodgate, R. (1997). The Saccharomyces cerevisiae *RAD30* gene, a homologue of *Escherichia coli dinB* and *umuC*, is DNA damage inducible and functions in a novel error-free postreplication repair mechanism. Genetics *147*, 1557-1568.

Stelter, P., and Ulrich, H.D. (2003). Control of spontaneous and damage-induced mutagenesis by SUMO and ubiquitin conjugation. Nature *425*, 188-191.

Torres-Ramos, C.A., Prakash, S., and Prakash, L. (2002). Requirement of *RAD5* and *MMS2* for postreplication repair of UV-damaged DNA in *Saccharomyces cerevisiae*. Mol. Cell. Biol. *22*, 2419-2426.
