# Supplementary material for: Hypermutability of Damaged Single-Strand DNA Formed at Double-Strand Breaks and Uncapped Telomeres in Yeast Saccharomyces cerevisiae
Source: PLoS Genet. 2008 Nov 21;4(11):e1000264. doi: 10.1371/journal.pgen.1000264 (PMC2577886; doi:10.1371/journal.pgen.1000264)
Supplement: Table S2 — Frequencies of spontaneous and MMS-induced can1 mutants associated with DSB-repair. (0.02 MB PDF) [file pgen.1000264.s002.pdf]

Table S2. Frequencies of spontaneous and MMS-induced *can1* mutants associated with DSB-repair

DSB position	Genotype	Time in galactose, hr	0.1% MMS, min	Frequency of *can1* mutants (x 10^4)				Transformation to Lys^+ or Ura^+		% MMS-survival (median)
				among transformants		in population				
				median	range [# of repeats] [1]	median	range	median frequency (x 10^7)	Fold over "no-DSB" control	
no	Wild type	6	0	NA	NA	0.11	(0.067-0.15)	5	NA	100
no	Wild type	6	15	NA	NA	0.17	(0.082-0.23)	29	NA	90
no	Wild type	6	30	NA	NA	0.29	(0.26-0.33)	47	NA	80
tel	Wild type	6	0	31	(20-41) [4]	0.40	(0.36-0.47)	1,357	NA	100
tel	Wild type	6	15	87	(76-111) [4]	0.58	(0.51-0.66)	1,480	NA	90
tel	Wild type	6	30	150	(130-180) [4]	0.51	(0.48-0.54)	619	NA	90
cen	Wild type	6	0	30	(29-33) [8]	0.91	(0.78-0.95)	4,747	1,048	100
cen	Wild type	6	15	210	(180-550) [8]	1.4	(0.47-1.7)	4,727	165	90
cen	Wild type	6	30	360	(200-520) [8]	1.6	(0.66-2.2)	7,514	161	80
cen	*rad30*	6	0	37	(29-41) [4]	1.7	(1.5-1.8)	4,105	907	100
cen	*rad30*	6	15	320	(280-430) [4]	2.0	(1.7-2.4)	2,361	82	90
cen	*rad30*	6	30	500	(430-540) [4]	2.5	(2.4-2.7)	2,667	57	80
cen	*rev3*	6	0	1.2	(0.79-1.5) [4]	1.1	(0.96-1.4)	4,299	949	100
cen	*rev3*	6	15	3.2	(2.3-3.7) [4]	0.56	(0.47-0.61)	1,204	42	80
cen	*rev3*	6	30	3.0	(1.9-4.2) [4]	0.48	(0.45-0.51)	1,562	33	70
TRP5 (Chr. VII)	Wild type	6	0	<0.28 {2}	[4]	<0.21 {3}	[4]	72,727	ND	100
TRP5 (Chr. VII)	Wild type	6	30	<0.28{2}	[4]	0.20	(0.17-0.24)	50,379	ND	90

[1] There were 4 to 8 repeats for the "no-DSB" controls.

See also footnotes 1, 2 and 5 to Table S1
