# Supplementary material for: Hypermutability of Damaged Single-Strand DNA Formed at Double-Strand Breaks and Uncapped Telomeres in Yeast Saccharomyces cerevisiae
Source: PLoS Genet. 2008 Nov 21;4(11):e1000264. doi: 10.1371/journal.pgen.1000264 (PMC2577886; doi:10.1371/journal.pgen.1000264)
Supplement: Table S3 — Frequencies of UV-induced Lys− mutants associated with uncapped telomere arrest. (0.02 MB PDF) [file pgen.1000264.s003.pdf]

Table S3. Frequencies of UV-induced Lys$^-$ mutants associated with uncapped telomere arrest

Mutations in addition to *cdc13-1*	Arrest[1] at 37°C, hr	UV-dose, J/m^2	Frequency of *lys2* mutants (x 10^4)		Frequency of Lys$^-$ (*LYS2*$^+$) mutants (x 10^4)		%UV-survival, median	Allelism of Lys$^-$ (*LYS2*$^+$) mutants [4]							
			median [number of mutants][2]	range	median [number of mutants]	range		*lys1* (IX, 19)	*lys4* (IV, 60)	*lys5* (VII, 215)	*lys7* (XIII, 348)	*lys9* (XIV, 69)	*lys12* (IX; 187)	*lys14* (IV, 510)	un-known
REV3 RAD30	0	0	<1 [0] [3]	NA	<2 [2]	NA	100	-	-	-	-	-	-	-	-
REV3 RAD30	6	0	<3 [2]	NA	<2 [1]	NA	100	-	-	-	-	-	-	-	-
REV3 RAD30	0	45	3 [21]	(0-8)	4 [30]	(2-18)	43	4	3	3	0	3	2	0	5
REV3 RAD30	6	45	182 [207]	(86-194)	5 [10]	(3-15)	37	2	2	1	0	1	0	0	4
rev3	0	0	<5 [0]	NA	<5 [0]	NA	100	-	-	-	-	-	-	-	-
rev3	6	0	<7 [0]	NA	<7 [0]	NA	100	-	-	-	-	-	-	-	-
rev3	0	45	<4 [0]	NA	<4 [0]	NA	10	-	-	-	-	-	-	-	-
rev3	6	45	<3 [3]	NA	<2 [1]	NA	33	-	-	-	-	-	-	-	-
rad30	0	0	<5 [0]	NA	<5 [0]	NA	100	-	-	-	-	-	-	-	-
rad30	6	0	<9 [1]	NA	<6 [0]	NA	100	-	-	-	-	-	-	-	-
rad30	0	45	3 [3]	(2-5)	15 [14]	(14-16)	15	1	3	2	0	1	1	0	4
rad30	6	45	132 [131]	(111-152)	26 [25]	(26-33)	29	6	3	1	0	0	2	0	11
rev3, rad30	0	0	<4 [0]	NA	<4 [0]	NA	100	-	-	-	-	-	-	-	-
rev3, rad30	6	0	<7 [0]	NA	<7 [0]	NA	100	-	-	-	-	-	-	-	-
rev3, rad30	0	45	<2 [0]	NA	<2 [0]	NA	4	-	-	-	-	-	-	-	-
rev3, rad30	6	45	<2 [0]	NA	<2 [0]	NA	11	-	-	-	-	-	-	-	-

Footnotes to Table S3.

[1] *cdc13-1* mutants were examined either as a G1 population after growing for 72 hr at 23°C or after additional incubation for 6 hr in fresh, rich YPDA medium at 37°C to develop G2-arrest associated with uncapped telomeres.

[2] [ ] shown in brackets are the total numbers of mutants obtained in 5 repeats of the experiment for the wild-type and in 2 repeats for translesion DNA polymerase mutants.

[3] Low frequencies were estimated using total number of mutants in all repeats of experiment assuming Poisson distribution;
only the upper limit of the 95% CI is shown.

[4] Mutants from the Lys^{-} (*LYS2*$^{+}$) category were tested for allelism with tester strains, each carrying a deletion of one of the 6 known *LYS*-genes. Chromosome assignment and distance (kb) from the nearest telomere is shown in parentheses.
"-", not tested
