# Supplementary material for: Hypermutability of Damaged Single-Strand DNA Formed at Double-Strand Breaks and Uncapped Telomeres in Yeast Saccharomyces cerevisiae
Source: PLoS Genet. 2008 Nov 21;4(11):e1000264. doi: 10.1371/journal.pgen.1000264 (PMC2577886; doi:10.1371/journal.pgen.1000264)
Supplement: Table S4 — Mutation spectrum in the category “can1 - no DSB; UV, 45 J/m2”. (0.02 MB PDF) [file pgen.1000264.s004.pdf]

Table S4. Mutation spectrum in the category "*can1* - no DSB; UV, 45 J/m^2"

Mutant #	Position in *CAN1* (coding strand)	Distance between adjacent mutations	WT base	Mutant base	Del/Add (-/+ # of nt)	WT sequence context	Mutant sequence context	Type of mutation	# of mutations in mutant
c01	529		T	A		GGTTTTCTtGGGCAATC	GGTTTTCTaGGGCAATC	sub	1
c02	1214		C	T		TGGTCTATcAAAGAACA	TGGTCTATtAAAGAACA	sub	1
c03	625		T	-	-1	GATTAGTAtTTTTTGGG	GATTAGTA-TTTTTGGG	indel	1
c04	572		CAT	TA-		GGCCAAGTcAtTCAATTTT	GGCCAAGTtA-TCAATTTT	compl	1
c07	1477		A	T		TTAAAGCTaAATTAATG	TTAAAGCTtAATTAATG	sub	1
c08	925		G	A		CTGCTGGTgAAGCTGCA	CTGCTGGTaAAGCTGCA	sub	1
c09	212				-55	GATGGCATaggtgatgaagatgaaggag<br>aagtacagaacgctgaagtgaagagaga<br>gcttaagCAAAGACA	GATGGCAT--------------------<br>----------------------------<br>-----CAAAGACA	55 nt deletion	1
c10	979		C	T		TTGTTTTCcGTATCTTA	TTGTTTTCtGTATCTTA	sub	1
c11	559		GT	TG		AACTTAGTgtAGTTGGCC	AACTTAGTtgAGTTGGCC	compl	1
c12	527		C	T		TTGGTTTTcTTGGGCAA	TTGGTTTTtTTGGGCAA	sub	1
c13	858		G	A		TTAGGTTGgGTTTCCTC	TTAGGTTGaGTTTCCTC	sub	1
c14	544		GCC	TTT		TCACTTTTgccCTGGAACT	TCACTTTTtttCTGGAACT	compl	1
c15	1226		T	-	-1	GAACAAGTtGGCTCCTA	GAACAAGT-GGCTCCTA	indel	1
c16	1488		C	-	-1	TTAATGCCcGGCTTGGC	TTAATGCC-GGCTTGGC	indel	1
c17	970		-	T	+1	AAAAAGTT-GTTTTCCG	AAAAAGTTtGTTTTCCG	indel	1
c18	311		C	G		TATTGGTAcAGGTCTTT	TATTGGTAgAGGTCTTT	sub	1
c19	627		G	A		ATTTTTTGgGTAATTAT	ATTTTTTGaGTAATTAT	sub	1
c20	707		T	A		CAAAGTTTtAGCCATTA	CAAAGTTTaAGCCATTA	sub	1
c21	1487		C	T		ATTAATGCcCGGCTTGG	ATTAATGCtCGGCTTGG	sub	1
c22	573		ATT	GT-		GCCAAGTCattCAATTTTG	GCCAAGTCgt-CAATTTTG	compl	1
c23	1089		ATT	---	-3	CCTTTATTattGCTATTGA	CCTTTATT---GCTATTGA	indel	1
c24	700		A	G		CTTCCATCaAAGTTTTA	CTTCCATCgAAGTTTTA	sub	1
c45	691		G	C		TCTGGGTCgCTTCCATC	TCTGGGTCcCTTCCATC	sub	1
c46	1557		CC	TA		CTTTTGCAccAAAATTCA	CTTTTGCAtaAAAATTCA	compl	1
c47	973		G	-	-1	AAAAAGTTgTTTTCCGT	AAAAAGTT-TTTTCCGT	indel	1
c48	1267		C	T		GTGGTGTTcCATACATT	GTGGTGTTtCATACATT	sub	1
c49	410		C	G		TTCTGTCAcGCAGTCCT	TTCTGTCAgGCAGTCCT	sub	1
c50	892		C	T		TCACATTTcAAGGTACT	TCACATTTtAAGGTACT	sub	1
c51	1018		G	A		TATTCATTgGACTTTTA	TATTCATTaGACTTTTA	sub	1
c52	527		C	T		TTGGTTTTcTTGGGCAA	TTGGTTTTtTTGGGCAA	sub	1
c54	686		GG	AA		GAGTTCTGggTCGCTTCC	GAGTTCTGaaTCGCTTCC	compl	1
c55	958		CCA	---	-3	TCCAAGAGccaTCAAAAAA	TCCAAGAG---TCAAAAAA	indel	1
c56	452		C	T		TGTTACATcCTCTTTCA	TGTTACATtCTCTTTCA	sub	1
c57	1487		C	T		ATTAATGCcCGGCTTGG	ATTAATGCtCGGCTTGG	sub	1
c59	226		A	-	-1	TGAAGATGaAGGAGAAG	TGAAGATG-AGGAGAAG	indel	1
c60	1007		ATT--	TTTTT		GGCTCTCTaTT--ATTCATTG	GGCTCTCTtTTttATTCATTG	compl	1
c61	263		T	C		GAGAGAGCtTAAGCAAA	GAGAGAGCcTAAGCAAA	sub	1
c62	297		GG	AA		CCCTTGGTggTACTATTG	CCCTTGGTaaTACTATTG	compl	1
c63	615		AGTATTTTTT	GGTATTTTT-		CATGGATTaGTATTTTTTtGGGTAATT	CATGGATTgGTATTTTT-GGGTAATT	compl	1
c64	263		T	C		GAGAGAGCtTAAGCAAA	GAGAGAGCcTAAGCAAA	sub	1
c65	1175		C	T		CGCAAATTcAAATATTT	CGCAAATTtAAATATTT	sub	1
c66	627		GG	AA		ATTTTTTGggTAATTATC	ATTTTTTGaaTAATTATC	compl	1
c67	827		GGATAAAAA	AGATAAAA-		ATATCTAAgGATAAAAaCGAAGGGA	ATATCTAAaGATAAAA-CGAAGGGA	compl	1
c53	838		G	A		ATAAAAACgAAGGGAGG	ATAAAAACaAAGGGAGG	sub	2
c53	926	88	A	G		TGCTGGTGaAGCTGCAA	TGCTGGTGgAGCTGCAA	sub	

See footnotes on the next page

Footnotes to Table S4 (aslo relates to Tables S5-S12)

Categories of mutants are explained in the text and in the legend to Figure 6.
Coordinates are given as in the coding strand of *CAN* , *URA3* starting from the first nucleotide of the *CAN1* and *URA3* ORFs as follows:

- simple base substitution (sub) –coordinate of a mutated base in the wild type sequence;
- simple insertions and deletions (indel)–coordinate of the last unchanged base in the wild type sequence;
- complex mutations (compl) –coordinate of the last unchanged base in the wild type sequence.
