# Supplementary material for: Hypermutability of Damaged Single-Strand DNA Formed at Double-Strand Breaks and Uncapped Telomeres in Yeast Saccharomyces cerevisiae
Source: PLoS Genet. 2008 Nov 21;4(11):e1000264. doi: 10.1371/journal.pgen.1000264 (PMC2577886; doi:10.1371/journal.pgen.1000264)
Supplement: Table S5 — Mutation spectrum in the category “can1 - DSB-cen; no UV”. (0.02 MB PDF) [file pgen.1000264.s005.pdf]

Table S5. Mutation spectrum in the category "*can1* - DSB-*cen*; no UV"

Mutant #	Position in *CAN1* (coding strand)	Distance between adjacent mutations	WT base	Mutant base	Del/Add (-/+ # of nt)	WT sequence context	Mutant sequence context	Type of mutation	# of mutations in mutant
61	670		G	A		AATATTACgGTGAATTC	AATATTACaGTGAATTC	sub	1
64	271		A	G		TTAAGCAAaGACATATT	TTAAGCAAgGACATATT	sub	1
65	449		C	G		CCCTGTTAcATCCTCTT	CCCTGTTAgATCCTCTT	sub	1
66	1279		T	-	-1	TGCAGTTTtCGTTACTG	TGCAGTTT-CGTTACTG	indel	1
69	1000		G	C		TCTACATTgGCTCTCTA	TCTACATTcGCTCTCTA	sub	1
71	880		C	-	-1	AACGCTGCcTTCACATT	AACGCTGC-TTCACATT	indel	1
73	959		C	A		TCCAAGAGcCATCAAAA	TCCAAGAGaCATCAAAA	sub	1
75	509		G	A		GGCCAATGgTTACATGT	GGCCAATGaTTACATGT	sub	1
76	673		G	A		ATTACGGTgAATTCGAG	ATTACGGTaAATTCGAG	sub	1
77	452		C	A		TGTTACATcCTCTTTCA	TGTTACATaCTCTTTCA	sub	1
90	1115		G	-	-1	GTACAAAGgTTTTGCCA	GTACAAAG-TTTTGCCA	indel	1
91	703		T	-	-1	CAAAGTTTtAGCCATTA	CAAAGTTT-AGCCATTA	indel	1
92	789		G	A		CGTTATTGgAGAAACCC	CGTTATTGaAGAAACCC	sub	1
93	422		G	C		GTCCTTGGgTGAAATGG	GTCCTTGGcTGAAATGG	sub	1
94	1208		G	A		TTTATTTGgTCTATCAA	TTTATTTGaTCTATCAA	sub	1
95	789		G	A		CGTTATTGgAGAAACCC	CGTTATTGaAGAAACCC	sub	1
96	1228		CTC	---	-3	CAAGTTGGctcCTAAATTC	CAAGTTGG---CTAAATTC	indel	1
99	673		G	A		ATTACGGTgAATTCGAG	ATTACGGTaAATTCGAG	sub	1
100	887		C	G		TGCCTTCAcATTTCAAG	TGCCTTCAgATTTCAAG	sub	1
102	430		G	C		GTGAAATGgCTACATTC	GTGAAATGcCTACATTC	sub	1
103	806		G	A		AGGTGCCTgGGGTCCAG	AGGTGCCTaGGGTCCAG	sub	1
104	1487		C	T		ATTAATGCcCGGCTTGG	ATTAATGCtCGGCTTGG	sub	1
105	515		T	G		TGGTTACAtGTATTGGT	TGGTTACAgGTATTGGT	sub	1
106	443		C	T		ATTCATCCcTGTTACAT	ATTCATCCtTGTTACAT	sub	1
107	1018		G	C		TATTCATTgGACTTTTA	TATTCATTcGACTTTTA	sub	1
108	928		G	A		CTGGTGAAgCTGCAAAC	CTGGTGAAaCTGCAAAC	sub	1
63	665		T	-	-1	TCAAATATtACGGTGAA	TCAAATAT-ACGGTGAA	indel	2
63	1162	497	CT	AA		CATTATTTctGCCGCAAA	CATTATTTaaGCCGCAAA	compl	

See footnotes to Table S4

The *CAN1* coding strand is complementary to unresected strand in the DSB-*cen* construct
