# Supplementary material for: Hypermutability of Damaged Single-Strand DNA Formed at Double-Strand Breaks and Uncapped Telomeres in Yeast Saccharomyces cerevisiae
Source: PLoS Genet. 2008 Nov 21;4(11):e1000264. doi: 10.1371/journal.pgen.1000264 (PMC2577886; doi:10.1371/journal.pgen.1000264)
Supplement: Table S6 — Mutation spectrum in the category “can1 - DSB-cen; UV, 20 J/m2”. (0.02 MB PDF) [file pgen.1000264.s006.pdf]

Table S6. Mutation spectrum in the category "*can1* - DSB-*cen*; UV, 20 J/m^2"

Mutant #	Position in *CAN1* (coding strand)	Distance between adjacent mutations	WT base	Mutant base	Del/Add (-/+ # of nt)	WT sequence context	Mutant sequence context	Type of mutation	# of mutations in mutant
120	619		T	-	-1	GTATTTTTtGGGTAATT	GTATTTTT-GGGTAATT	indel	1
122	894		A	T		ACATTTCAaGGTACTGA	ACATTTCAtGGTACTGA	sub	1
123	941		A	-	-1	CCCAGAAAaTCCGTTCC	CCCAGAAA-TCCGTTCC	indel	1
125	252		A	-	-1	TGAAGTGAaGAGAGAGC	TGAAGTGA-GAGAGAGC	indel	1
126	703		-	T	1	AAAGTTTT-AGCCATTA	AAAGTTTTtAGCCATTA	indel	1
127	548		GG	AA		TTTGCCCTggAACTTAGT	TTTGCCCTaaAACTTAGT	compl	1
130	685		G	T		GAATTCGAgTTCTGGGT	GAATTCGAtTTCTGGGT	sub	1
133	424		G	-	-1	CCTTGGGTgAAATGGCT	CCTTGGGT-AAATGGCT	indel	1
135	669		GG	AA		AATATTACggTGAATTCG	AATATTACaaTGAATTCG	compl	1
136	1314		ATGG	TTG-		TGGCTTACaTGgAGACATCT	TGGCTTACtTG-AGACATCT	compl	1
137	970		G	-	-1	TCAAAAAAgTTGTTTTC	TCAAAAAA-TTGTTTTC	indel	1
139	228		G	-	-1	AGATGAAGgAGAAGTAC	AGATGAAG-AGAAGTAC	indel	1
142	380		A	-	-1	TCATATTTaTTTATGGG	TCATATTT-TTTATGGG	indel	1
219	1538		A	G		CATTATTCaAGGTTTCA	CATTATTCgAGGTTTCA	sub	1
221	741		T	A		TGTTTTTGtATGGTTTG	TGTTTTTGaATGGTTTG	sub	1
233	943		A	T		ACCCCAGAaAATCCGTT	ACCCCAGAtAATCCGTT	sub	1
241	585		G	A		CAATTTTGgACGTACAA	CAATTTTGaACGTACAA	sub	1
121	271		A	G		TTAAGCAAaGACATATT	TTAAGCAAgGACATATT	sub	2
121	936	665	C	A		GCTGCAAAcCCCAGAAA	GCTGCAAAaCCCAGAAA	sub	
128	625		GG	AA		TATTTTTTggGTAATTAT	TATTTTTTaaGTAATTAT	compl	2
128	1435	810	A	T		AAGCTTTGaAATACCGT	AAGCTTTGtAATACCGT	sub	
129	132		G	A		AAAGATGAgAAAAGTAA	AAAGATGAaAAAAGTAA	sub	2
129	1234	1102	A	T		TGGCTCCTaAATTCCTG	TGGCTCCTtAATTCCTG	sub	
229	611		G	A		GGCGGCATgGATTAGTA	GGCGGCATaGATTAGTA	sub	2
229	665	54	T	-	-1	TCAAATATtACGGTGAA	TCAAATAT-ACGGTGAA	indel	
232	26		A	C		AGACGCCGaCATAGAGG	AGACGCCGcCATAGAGG	sub	2
232	548	522	GG	AA		TTTGCCCTggAACTTAGT	TTTGCCCTaaAACTTAGT	compl	
138	789		A	G		GTTATTGGaGAAA-CCCAGGTG	GTTATTGGgGAAAaCCCAGGTG	compl	2
138	906	117	A	T		ACTGAACTaGTTGGTAT	ACTGAACTtGTTGGTAT	sub	
141	255		G	A		GCTGAAGTgAAGAGAGA	GCTGAAGTaAAGAGAGA	sub	3
141	687	432	G	A		GAGTTCTGgGTCGCTTC	GAGTTCTGaGTCGCTTC	sub	
141	1405	718	A	T		TTATCTCAaTCTCGCAC	TTATCTCAtTCTCGCAC	sub	
220	275		A	T		GCAAAGACaTATTGGTA	GCAAAGACtTATTGGTA	sub	3
220	1106	831	C	T		TGAGAACTcTGGTACAA	TGAGAACTtTGGTACAA	sub	
220	1512	406	A	G		GCGGCCACaTTTATGAC	GCGGCCACgTTTATGAC	sub	

See footnotes to Table S4

The *CAN1* coding strand is complementary to unresected strand in the DSB-*cen* construct
