# Supplementary material for: Hypermutability of Damaged Single-Strand DNA Formed at Double-Strand Breaks and Uncapped Telomeres in Yeast Saccharomyces cerevisiae
Source: PLoS Genet. 2008 Nov 21;4(11):e1000264. doi: 10.1371/journal.pgen.1000264 (PMC2577886; doi:10.1371/journal.pgen.1000264)
Supplement: Table S7 — Mutation spectrum in the category “can1 - DSB-cen; UV 45 J/m2”. (0.02 MB PDF) [file pgen.1000264.s007.pdf]

Table S7. Mutation spectrum in the category "*can1* - DSB-*cen* ; UV 45 J/m^2"

Mutant #	Position in *CAN1* (coding strand)	Distance between adjacent mutations	WT base	Mutant base	Del/Add (-/+ # of nt)	WT sequence context	Mutant sequence context	Type of mutation	# of mutations in mutant
317	177		A	G		ACGAGAGTaAATGGCGA	ACGAGAGTgAATGGCGA	sub	1
319	290		C	A		TATGATTGcCCTTGGTG	TATGATTGaCCTTGGTG	sub	1
320	941		A	-	-1	CCCAGAAAaTCCGTTCC	CCCAGAAA-TCCGTTCC	indel	1
328	1217		-	G	+1	TATCAAAG-AACAAGTT	TATCAAAGgAACAAGTT	indel	1
331	1679		A	T		AGATGTCGaCATCGATT	AGATGTCGtCATCGATT	sub	1
325	670		GG	AA		AATATTACggTGAATTCG	AATATTACaaTGAATTCG	compl	1
321	307		G	A		GTACTATTggGTACAGGT	GTACTATTaaGTACAGGTC	compl	1
322	296		G	T		TGCCCTTGgTGGTACTA	TGCCCTTGtTGGTACTA	sub	2
322	1018	722	G	A		TATTCATTgGACTTTTA	TATTCATTaGACTTTTA	sub	
329	1179		T	A		AATTCAAAtATTTACGT	AATTCAAAaATTTACGT	sub	2
329	1541	362	G	T		TATTCAAGgTTTCACGG	TATTCAAGtTTTCACGG	sub	
332	685		GG	AA		CGAGTTCTggGTCGCTTC	CGAGTTCTaaGTCGCTTC	compl	2
332	1418	733	G	A		GCACATCAgATTTATGC	GCACATCAaATTTATGC	sub	
326	785		A	T		ATTCCGTTaTTGGAGAA	ATTCCGTTtTTGGAGAA	sub	3
326	997	212	A	T		CCTTCTACaTTGGCTCT	CCTTCTACtTTGGCTCT	sub	
326	1175	178	A	-	-1	AAATTCAAaTATTTACG	AAATTCAA-TATTTACG	indel	

See footnotes to Table S4

The *CAN1* coding strand is complementary to unresected strand in the DSB-*cen* construct
