# Supplementary material for: Hypermutability of Damaged Single-Strand DNA Formed at Double-Strand Breaks and Uncapped Telomeres in Yeast Saccharomyces cerevisiae
Source: PLoS Genet. 2008 Nov 21;4(11):e1000264. doi: 10.1371/journal.pgen.1000264 (PMC2577886; doi:10.1371/journal.pgen.1000264)
Supplement: Table S8 — Mutation spectrum in the category “can1 (from can1 ura3 set) - DSB-cen; UV, 45 J/m2”. (0.02 MB PDF) [file pgen.1000264.s008.pdf]

Table S8. Mutation spectrum in the category "*can1* (from *can1 ura3* set) - DSB-*cen* ; UV, 45 J/m^2"

Mutant #	Position in *CAN1* (coding strand)	Distance between adjacent mutations	WT base	Mutant base	Del/Add (-/+ # of nt)	WT sequence context	Mutant sequence context	Type of mutation	# of mutations in mutant
2	584		G	A		TCAATTTTgGACGTACA	TCAATTTTaGACGTACA	sub	1
4	687		G	A		GAGTTCTGgGTCGCTTC	GAGTTCTGaGTCGCTTC	sub	1
5	1143		A	-	-1	ACGCTGTTaTCTTAACA	ACGCTGTT-TCTTAACA	indel	1
8	130		G	T		TAAAAGATgAGAAAAGT	TAAAAGATtAGAAAAGT	sub	1
9	926		A	T		TGCTGGTGaAGCTGCAA	TGCTGGTGtAGCTGCAA	sub	1
10	612		G	A		GCGGCATGgATTAGTAT	GCGGCATGaATTAGTAT	sub	1
16	1208		G	A		TTTATTTGgTCTATCAA	TTTATTTGaTCTATCAA	sub	1
19	1179		T	A		AATTCAAAtATTTACGT	AATTCAAAaATTTACGT	sub	1
20	612		G	A		GCGGCATGgATTAGTAT	GCGGCATGaATTAGTAT	sub	1
21	271		A	T		TTAAGCAAaGACATATT	TTAAGCAAtGACATATT	sub	1
23	673		G	A		ATTACGGTgAATTCGAG	ATTACGGTaAATTCGAG	sub	1
24	963		A	-	-1	ATCAAAAAaGTTGTTTT	ATCAAAAA-GTTGTTTT	indel	1
25	1171		A	T		CTGCCGCAaATTCAAAT	CTGCCGCAtATTCAAAT	sub	1
27	180		G	-	-1	AGTAAATGgCGAGGATA	AGTAAATG-CGAGGATA	indel	1
6	299		G	T		CCTTGGTGgTACTATTG	CCTTGGTGtTACTATTG	sub	2
6	1223	924	A	T		AAAGAACAaGTTGGCTC	AAAGAACAtGTTGGCTC	sub	
13	1037		A	G		TCCATACAaTGACCCTA	TCCATACAgTGACCCTA	sub	2
13	1087	50	A	T		CTCCCTTTaTTATTGCT	CTCCCTTTtTTATTGCT	sub	
17	135		A	T		GATGAGAAaAGTAAAGA	GATGAGAAtAGTAAAGA	sub	2
17	530	395	GG	AA		TTTTCTTGggCAATCACT	TTTTCTTGaaCAATCACT	compl	
26	362		G	A		CCCAGTGGgCGCTCTTA	CCCAGTGGaCGCTCTTA	sub	2
26	1706	1344	A	G		AGACATTGaGGCAATTG	AGACATTGgGGCAATTG	sub	
12	673		G	A		ATTACGGTgAATTCGAG	ATTACGGTaAATTCGAG	sub	3
12	1208	535	G	T		TTTATTTGgTCTATCAA	TTTATTTGtTCTATCAA	sub	
12	1698	490	A	G		GATAGAAGaGACATTGA	GATAGAAGgGACATTGA	sub	
15	965		-	G	+1	GCCATCAA-AAAAGTTG	GCCATCAAgAAAAGTTG	indel	3
15	1103	138	A	T		TATTGAGAaCTCTGGTA	TATTGAGAtCTCTGGTA	sub	
15	1203	100	A	T		CGTATTTTaTTTGGTCT	CGTATTTTtTTTGGTCT	sub	
28	829		ATAAAAA	TTAAAAA-		ATCTAAGGaTAAAAaCGAAGGGA	ATCTAAGGtTAAAA-CGAAGGGA	compl	3
28	1115	286	A	G		TGGTACAAaGGTTTTGC	TGGTACAAgGGTTTTGC	sub	
28	1129	14	A	C		TGCCACATaTCTTCAAC	TGCCACATcTCTTCAAC	sub	

See footnotes to Table S4

The *CAN1* coding strand is complementary to unresected strand in the DSB-*cen* construct
