# Supplementary material for: Hypermutability of Damaged Single-Strand DNA Formed at Double-Strand Breaks and Uncapped Telomeres in Yeast Saccharomyces cerevisiae
Source: PLoS Genet. 2008 Nov 21;4(11):e1000264. doi: 10.1371/journal.pgen.1000264 (PMC2577886; doi:10.1371/journal.pgen.1000264)
Supplement: Table S9 — Mutation spectrum in the category “ura3 (from can1 ura3 set) - DSB-cen; UV, 45 J/m2”. (0.02 MB PDF) [file pgen.1000264.s009.pdf]

Table S9. Mutation spectrum in the category "*ura3* (from *can1 ura3* set) - DSB-*cen*; UV, 45 J/m^2"

Mutant #	Position in *URA3* (coding strand)	Distance between adjacent mutations	WT base	Mutant base	Del/Add (-/+ # of nt)	WT sequence context	Mutant sequence context	Type of mutation	# of mutations in mutant
3	438		CTTTTGAT	TTTTTGAA		CTAGAGGCcTTTTGAtGTTAGCAG	CTAGAGGCtTTTTGAaGTTAGCAG	compl	1
5	534		T	C		AAAGATTTtGTTATCGG	AAAGATTTcGTTATCGG	sub	1
6	543		--	TT	+2	ATCGGCTT--TATTGCTC	ATCGGCTTttTATTGCTC	indel	1
8	173		T	C		TTGTTTACtAAAAACAC	TTGTTTACcAAAAACAC	sub	1
11	546		TTG	AAT		CGGCTTTAttgCTCAAAGA	CGGCTTTAaatCTCAAAGA	compl	1
13	258		-	A	+1	ACAATTTT-TTACTCTT	ACAATTTTaTTACTCTT	indel	1
14	335		T	A		ATACAGAAtAGCAGAAT	ATACAGAAaAGCAGAAT	sub	1
15	553		C	T		TTATTGCTcAAAGAGAC	TTATTGCTtAAAGAGAC	sub	1
16	200		--	TT	+2	TGATTTTT--CCATGGAG	TGATTTTTttCCATGGAG	indel	1
17	360		T	A		ATTACGAAtGCACACGG	ATTACGAAaGCACACGG	sub	1
18	389		T	A		AGGTATTGtTAGCGGTT	AGGTATTGaTAGCGGTT	sub	1
19	168		T	A		AAAATTTGtTTACTAAA	AAAATTTGaTTACTAAA	sub	1
20	373		T	-	-1	CGGTGTGGtGGGCCCAG	CGGTGTGG-GGGCCCAG	indel	1
22	104		C	T		GTGTGCTTcATTGGATG	GTGTGCTTtATTGGATG	sub	1
23	605		C	A		TATGACACcCGGTGTGG	TATGACACaCGGTGTGG	sub	1
27	230		T	C		TAAGCCGCtAAAGGCAT	TAAGCCGCcAAAGGCAT	sub	1
28	2		T	A		ATAAATCAtGTCGAAAG	ATAAATCAaGTCGAAAG	sub	1
30	246		A	-	-1	ATCCGCCAaGTACAATT	ATCCGCCA-GTACAATT	indel	1
33	651		T	G		CAACAGTAtAGAACCGT	CAACAGTAgAGAACCGT	sub	1
36	254		T	-	-1	CAATTTTTtACTCTTCG	CAATTTTT-ACTCTTCG	indel	1
39	698		T	A		TATTATTGtTGGAAGAG	TATTATTGaTGGAAGAG	sub	1
42	604		CC	TT		TATGACACccGGTGTGGG	TATGACACttGGTGTGGG	compl	1
43	356		C	A		AGACATTAcGAATGCAC	AGACATTAaGAATGCAC	sub	1
44	623		C	-	-1	TTAGATGAcAAGGGAGA	TTAGATGA-AAGGGAGA	indel	1
45	242		C	T		GGCATTATcCGCCAAGT	GGCATTATtCGCCAAGT	sub	1
47	221		T	A		GGGCACAGtTAAGCCGC	GGGCACAGaTAAGCCGC	sub	1
48	62		T	C		TGCCAAGCtATTTAATA	TGCCAAGCcATTTAATA	sub	1
49	698		T	A		TATTATTGtTGGAAGAG	TATTATTGaTGGAAGAG	sub	1
50	200		-	T	+1	TGATTTTT-CCATGGAG	TGATTTTTtCCATGGAG	indel	1
56	636		T	-	-1	AGACGCATtGGGTCAAC	AGACGCAT-GGGTCAAC	indel	1
57	230		T	C		TAAGCCGCtAAAGGCAT	TAAGCCGCcAAAGGCAT	sub	1
2	159		A	-	-1	GTCCCAAAaTTTGTTTA	GTCCCAAA-TTTGTTTA	indel	2
2	313	154	T	A		AATTGCAGtACTCTGCG	AATTGCAGaACTCTGCG	sub	
4	537		T	C		GATTTTGTtATCGGCTT	GATTTTGTcATCGGCTT	sub	2
4	614	77	--	TT	+2	GTGGGTTT--AGATGACA	GTGGGTTTttAGATGACA	indel	
51	419		C	-	-1	AGAAGTAAcAAAGGAAC	AGAAGTAA-AAAGGAAC	indel	2
51	473	54	C	T		CAAGGGCTcCCTATCTA	CAAGGGCTtCCTATCTA	sub	

See footnotes to Table S4

The *URA3* coding strand corresponds to unresected strand in the DSB-*cen* construct
