# Supplementary material for: Hypermutability of Damaged Single-Strand DNA Formed at Double-Strand Breaks and Uncapped Telomeres in Yeast Saccharomyces cerevisiae
Source: PLoS Genet. 2008 Nov 21;4(11):e1000264. doi: 10.1371/journal.pgen.1000264 (PMC2577886; doi:10.1371/journal.pgen.1000264)
Supplement: Table S10 — Mutation spectrum in the category “can1 - DSB-tel; UV, 45 J/m2”. (0.02 MB PDF) [file pgen.1000264.s010.pdf]

Table S10. Mutation spectrum in the category "*can1* - DSB-*tel*; UV, 45 J/m^2"

Mutant #	Position in *CAN1* (coding strand)	Distance between adjacent mutations	WT base	Mutant base	Del/Add (-/+ # of nt)	WT sequence context	Mutant sequence context	Type of mutation	# of mutations in mutant
T55	1632		C	T		TCTTATTTcAATGCATA	TCTTATTTtAATGCATA	sub	1
T56	1185		C	A		AATATTTAcGTTGGTTC	AATATTTAaGTTGGTTC	sub	1
T58	977		T	C		AGTTGTTTtCCGTATCT	AGTTGTTTcCCGTATCT	sub	1
T59	577		C	T		AAGTCATTcAATTTTGG	AAGTCATTtAATTTTGG	sub	1
T49	519		T	A		TACATGTAtTGGTTTTC	TACATGTAaTGGTTTTC	sub	2
T49	1071	552	T	A		TCCTACGTtTCTACTTC	TCCTACGTaTCTACTTC	sub	
T50	900		T	C		CAAGGTACtGAACTAGT	CAAGGTACcGAACTAGT	sub	2
T50	1211	311	T	C		ATTTGGTCtATCAAAGA	ATTTGGTCcATCAAAGA	sub	
T51	419		T	A		GCAGTCCTtGGGTGAAA	GCAGTCCTaGGGTGAAA	sub	2
T51	540	121	T	C		GCAATCACtTTTGCCCTG	GCAATCACcTTTGCCCTG	sub	
T52	1029		T	G		CGCCGACAtAGAGGAGA	CGCCGACAgAGAGGAGA	sub	2
T52	1442	413	C	A		CATTCATCcCTGTTACA	CATTCATCaCTGTTACA	sub	
T53	264		T	C		AGAGAGCTtAAGCAAAG	AGAGAGCTcAAGCAAAG	sub	2
T53	553	289	C	T		CCCTGGAAcTTAGTGTA	CCCTGGAAtTTAGTGTA	sub	
T54	1163		C	T		CATTATTTcTGCCGCAA	CATTATTTtTGCCGCAA	sub	2
T54	1202	39	T	C		CCGTATTTtATTTGGTC	CCGTATTTcATTTGGTC	sub	
T57	851		T	A		GAGGTTCTtAGGTTGGG	GAGGTTCTaAGGTTGGG	sub	2
T57	1455	604	C	T		TACATCCTcTTTCACAG	TACATCCTtTTTCACAG	sub	
T60	402		T	A		TTGGCATAtTCTGTCAC	TTGGCATAaTCTGTCAC	sub	2
T60	1058	656	C	T		AACACAATcTACTTCCT	AACACAATtTACTTCCT	sub	
T61	99		C	T		CAAACACAcCACAGACG	CAAACACAtCACAGACG	sub	2
T61	937	838	CC	AT		CTGCAAACccCAGAAAAT	CTGCAAACatCAGAAAAT	compl	
T62	862		-	T	+1	TTGGGTTT-CCTCTTTG	TTGGGTTTtCCTCTTTG	indel	2
T62	1551	689	T	C		TTCACGGCtTTTGCACC	TTCACGGCcTTTGCACC	sub	
T63	836		A	T		GGATAAAAaCGAAGGGA	GGATAAAAtCGAAGGGA	sub	2
T63	1179	343	T	A		AATTCAAAtATTTACGT	AATTCAAAaATTTACGT	sub	
T64	530		G	T		GTTTTCTTgGGCAATCA	GTTTTCTTtGGCAATCA	sub	2
T64	1025	495	-	T	+1	GGACTTTT-AGTTCCAT	GGACTTTTtAGTTCCAT	indel	

See footnotes to Table S4

The *CAN1* coding strand corresponds to unresected strand in the DSB-*tel* construct
