# Supplementary material for: Hypermutability of Damaged Single-Strand DNA Formed at Double-Strand Breaks and Uncapped Telomeres in Yeast Saccharomyces cerevisiae
Source: PLoS Genet. 2008 Nov 21;4(11):e1000264. doi: 10.1371/journal.pgen.1000264 (PMC2577886; doi:10.1371/journal.pgen.1000264)
Supplement: Table S11 — Mutation spectra in the category “subtelomeric lys2, 23°C (no arrest) UV, 45 J/m2”. (0.04 MB PDF) [file pgen.1000264.s011.pdf]

Table S11. Mutation spectrum in the category "subtelomeric *lys2* , 23oC (no arrest) UV, 45 J/m^2"

Mutant number	Position in *chromosome V (distance from telomere)*	Distance between adjacent mutations	WT base	Mutant base	Del/Add (-/+ # of nt)	WT sequence context	Mutant sequence context	Type of mutation	# of *lys2* mutations in mutant	# of *npr2* mutations in mutant[1]
m2	4636		T	A		TTTTGATTtAACTTCGA	TTTTGATTaAACTTCGA	sub	1	0
m4	5088		G	A		TACCTTCGgAACCAGAT	TACCTTCGaAACCAGAT	sub	1	0
m7	4685		A	T		CCGTACATaTTAACAAT	CCGTACATtTTAACAAT	sub	1	0
m114	4954		G	A		ATCTCTTTgAATTGGAT	ATCTCTTTaAATTGGAT	sub	1	
m118	2785		C	T		TAAGAAATcATCTGTGT	TAAGAAATtATCTGTGT	sub	1	
m120	5293		G	A		TACTAGTTgATCCAATT	TACTAGTTaATCCAATT	sub	1	
m122	4926		A	T		GGGCACCTaAAAATAAT	GGGCACCTtAAAATAAT	sub	1	
m124	6080		C	T		TTAAATGAcCACGTTGG	TTAAATGAtCACGTTGG	sub	1	
m127	3647		-	A	+1	GATACTTG-AAAATTGT	GATACTTGaAAAATTGT	indel	1	
m131	6303		A	T		CATGTGGTaACACTGAA	CATGTGGTtACACTGAA	sub	1	0
m132	2542		T	A		CTTCCATTtAGAATAGC	CTTCCATTaAGAATAGC	sub	1	
m135	4246		G	A		GAACCCACgAATTTTAA	GAACCCACaAATTTTAA	sub	1	
m137	5039		A	T		TTGAAATAaTAAGCCAA	TTGAAATAtTAAGCCAA	sub	1	0
m138	2989		T	A		AACAAGTTtATCTGACA	AACAAGTTaATCTGACA	sub	1	0
m140	3995		-	A	+1	TCTTTAAG-AAAGTCCT	TCTTTAAGaAAAGTCCT	indel	1	
m142	4388		A	T		TTATCCAAaTAATTCCA	TTATCCAAtTAATTCCA	sub	1	
m145	3013		C	A		GTAGTATTcAGTGTCAA	GTAGTATTaAGTGTCAA	sub	1	0
m149	3916		T	C		ATCAACTTtACCATTTG	ATCAACTTcACCATTTG	sub	1	0
m150	3545		A	T		TCTTCCGCaTAATTTGC	TCTTCCGCtTAATTTGC	sub	1	0
m152	4246		G	A		GAACCCACgAATTTTAA	GAACCCACaAATTTTAA	sub	1	

[1] Number of *npr2* mutations found in the variants, in which *NPR2* ORF has been sequenced

See also footnotes to Table S4
