# Supplementary material for: Hypermutability of Damaged Single-Strand DNA Formed at Double-Strand Breaks and Uncapped Telomeres in Yeast Saccharomyces cerevisiae
Source: PLoS Genet. 2008 Nov 21;4(11):e1000264. doi: 10.1371/journal.pgen.1000264 (PMC2577886; doi:10.1371/journal.pgen.1000264)
Supplement: Table S12 — Mutation spectrum in the category “subtelomeric lys2, 37°C (arrest) UV, 45 J/m2”. (0.04 MB PDF) [file pgen.1000264.s012.pdf]

Table S12. Mutation spectrum in the category "subtelomeric *lys2* , 37oC (arrest) UV, 45 J/m^2"

Mutant number	Position in *chromo-some V (distan-ce from telo-mere)*	Distance between adjacent mutations	WT base	Mutant base	Del/Add (-/+ # of nt)	WT sequence context	Mutant sequence context	Type of mutation	# of *lys2* mutations in mutant	# of *npr2* mutati-ons in mutant[2]
m30	4765		A	-	-1	AAGAACGCaTGATGTAA	AAGAACGC-TGATGTAA	indel	1	
m37	5503		-	T	+1	TTAATCAA-ATAATGGG	TTAATCAAtATAATGGG	indel	1	
m35	5293		G	A		TACTAGTTgATCCAATT	TACTAGTTaATCCAATT	sub	1	
m40	3545		A	T		TCTTCCGCaTAATTTGC	TCTTCCGCtTAATTTGC	sub	1	
m43	5103		G	A		ATGTGAAAgATAGGGTT	ATGTGAAAaATAGGGTT	sub	1	
m47	6188		A	-		TCAAAGCGaCAGCGTAT	TCAAAGCG-CAGCGTAT	indel	1	
m48	5934		-,A	G,G		TTCCAAAA-aGGCCAAAC	TTCCAAAAggGGCCAAAC	compl	1	
m77	4249		T	A		CCCACGAAtTTTAACTT	CCCACGAAaTTTAACTT	sub	2	
m77	5171	922	A	G		TCATATGGaGCCAAGAC	TCATATGGgGCCAAGAC	sub		
m79	2454		AG	TA		TTCAGGTAagTTGTCTAA	TTCAGGTAtaTTGTCTAA	compl	2	
m79	6007	3553	G	-	-1	TCGTCAAAgGAAAAATT	TCGTCAAA-GAAAAATT	indel		
m84	3394		C	A		TAACAAATcTGCAAGGA	TAACAAATaTGCAAGGA	sub	2	
m84	5457	2063	G	A		CCCTAGAAgAATAGATC	CCCTAGAAaAATAGATC	sub		
m85	2678		ACA	---	-3	ACGTAGCAacaACAACACG	ACGTAGCA---ACAACACG	indel	2	
m85	5108	2430	G	A		AAAGATAGgGTTGGGTT	AAAGATAGaGTTGGGTT	sub		
m87	3139		A	G		ATAAACCCaGTGAACTA	ATAAACCCgGTGAACTA	sub	2	
m87	4584	1445	G	A		TACCAGCAgGCATGACA	TACCAGCAaGCATGACA	sub		
m21	2513		A	G		CTGTCAATaACAGACGC	CTGTCAATgACAGACGC	sub	2	
m21	3130	617	A	-	-1	GCATATGGaTAAACCCA	GCATATGG-TAAACCCA	indel		
m28	4180		T	A		AGTAATGTtTTCTCTTA	AGTAATGTaTTCTCTTA	sub	2	
m28	4424	244				TTTTCAAC-AAACCAGTTgTtCACAAATT	TTTTCAACtAAACCAGTTcTcCACAAATT	compl		
m29	3081		A	C		CTAAGCTCaTAACATTG	CTAAGCTCcTAACATTG	sub	2	
m29	3581	500	A	G		TCACCTTGaGATGATCC	TCACCTTGgGATGATCC	sub		
m31	3985		T	A		AGCCAATCtTTTCTTTA	AGCCAATCaTTTCTTTA	sub	2	
m31	4964	979	A	T		ATTGGATCaTGTGCAAT	ATTGGATCtTGTGCAAT	sub		
m34	4554		A	G		CAACTAGTaGCTGAACG	CAACTAGTgGCTGAACG	sub	2	
m34	5821	1267	G	-	-1	TACAGTAAgCTGTTATA	TACAGTAA-CTGTTATA	indel		
m38	3211		A	-	-1	TCTGAAAGaCCAAATTG	TCTGAAAG-CCAAATTG	indel	2	
m38	3925	714	G	C		ACCATTTGgATTCAATG	ACCATTTGcATTCAATG	sub		
m39	2782					TTCAAAAA-TCTCAATAAGaAATCATCT	TTCAAAAAcTCTCAATAAG-AATCATCT	compl	2	
m39	4386	1604	A	T		CCTTATCCaAATAATTC	CCTTATCCtAATAATTC	sub		
m41	3890		A	-	-1	GAGTTGGGaATTGAAGT	GAGTTGGG-ATTGAAGT	indel	2	
m41	5396	1506	A	G		ATAACTGAaAAGGTTGC	ATAACTGAgAAGGTTGC	sub		
m44	2300		C	-	-1	GTAAAAATcCAACCTTG	GTAAAAAT-CAACCTTG	indel	2	
m44	4860	2560	A	T		ACTTACTCaTCCATTCC	ACTTACTCtTCCATTCC	sub		
m46	5715		A	T		ATGCGGTGaTCAGAGAG	ATGCGGTGtTCAGAGAG	sub	2	0
m46	6357	642	A	C		CGTTAGTCaTTAGTGAG	CGTTAGTCcTTAGTGAG	sub		
m19	5553		G	A		AAGTGAAAgAACGGGAC	AAGTGAAAaAACGGGAC	sub	2	
m19	6078	525	A	-	-1	ATTAAATGaCCACGTTG	ATTAAATG-CCACGTTG	indel		
m78	3666		A	T		CCAATGGTaAATCAACT	CCAATGGTtAATCAACT	sub	3	0
m78	6311	2645	A	G		AACACTGAaAGAGTTGG	AACACTGAgAGAGTTGG	sub		
m78	6406	95	A	-	-1	TTTCACTGaAATTCCAG	TTTCACTG-AATTCCAG	indel		
m83(N)[1]	601		A	T		AATCGGAAaGAGATCCA	AATCGGAAtGAGATCCA	sub		2
m83(N)	851	250	AT	TG		TACTCCGAatGCCTCATA	TACTCCGAtgGCCTCATA	compl		
m83	5375	4524	A	T		GCTGGGGGaTATGCAGG	GCTGGGGGtTATGCAGG	sub	3	
m83	5831	456	A, A	T, G		TGTTATAAaTTAaGTTCAAAA	TGTTATAAtTTAgGTTCAAAA	compl		
m83	6219	388	G	A		TATCATGAgGCACATCG	TATCATGAaGCACATCG	sub		
m22	2547		T	A		ATTTAGAAtAGCTTTCG	ATTTAGAAaAGCTTTCG	sub	3	0
m22	5630	3083	-	A	+1	TGTCCTGG-AAAATGTC	TGTCCTGGaAAAATGTC	indel		
m22	5890	260	A	C		GTCCACTAaATGATGCT	GTCCACTAcATGATGCT	sub		

**Mutant number**	**Position in *chromo-some V (distan-ce from telo-mere)***	**Distance between adjacent mutations**	**WT base**	**Mutant base**	**Del/Add (-/+ # of nt)**	**WT sequence context**	**Mutant sequence context**	**Type of mutation**	**# of *lys2* mutations in mutant**	**# of *npr2* mutati-ons in mutant[2]**
m33(N)	525		G	-	-1	CTCCCTACgAACCAGCT	CTCCCTAC-AACCAGCT	indel		2
m33(N)	1965	1440	A	T		AATGCCGAaACATTTGA	AATGCCGAtACATTTGA	sub		
m33	3780	1815	A	T		TGGTAGGTaATATACTT	TGGTAGGTtATATACTT	sub	3	
m33	4205	425	A	T		TGTTGGGAaATGTGCGT	TGTTGGGAtATGTGCGT	sub		
m33	4494	289	A	T		GAACATAAaTCTCACCT	GAACATAAtTCTCACCT	sub		
m42	3997		A	T		CTTTAAGAaAGTCCTGA	CTTTAAGAtAGTCCTGA	sub	3	0
m42	5049	1052	-	A	+1	AGCCAAGG-AAAAATGT	AGCCAAGGaAAAAATGT	indel		
m42	5240	191	A	T		GCGATGGAaTTGATTCT	GCGATGGAtTTGATTCT	sub		
m36(N)	865		A	T		TCATACCAaTTGATGAG	TCATACCAtTTGATGAG	sub		1
m36	3376	2511	G	A		GTTCTTTGgAGAACGTC	GTTCTTTGaAGAACGTC	sub	4	
m36	3452	76	G	-	-1	TAATTGTTgTTTTTCCT	TAATTGTT-TTTTTCCT	indel		
m36	4389	937	T	A		TATCCAAAtAATTCCAG	TATCCAAAaAATTCCAG	sub		
m36	4610	221	A	C		TTTTTCAAaAAGTTTGG	TTTTTCAAcAAGTTTGG	sub		
m45(N)	1045		A	G		GCATTGCCaAAATTTCT	GCATTGCCgAAATTTCT	sub		1
m45	2783	1738	A	T		AATAAGAAaTCATCTGT	AATAAGAAtTCATCTGT	sub	4	
m45	3716	933	G	A		GTAGCCAAgATAGAATG	GTAGCCAAaATAGAATG	sub		
m45	4799	1083	G	A		GTAGCTTGgGCAGTAAG	GTAGCTTGaGCAGTAAG	sub		
m45	6091	1292	G	A		CGTTGGTTgAATATTGA	CGTTGGTTaAATATTGA	sub		
m18	3039		A	C		TGGAGGAAaCAAAGTCA	TGGAGGAAcCAAAGTCA	sub	4	0
m18	3168	129	A	T		GGATAATTaTATCAACT	GGATAATTtTATCAACT	sub		
m18	4202	1034	G	T		GGATGTTGgGAAATGTG	GGATGTTGtGAAATGTG	sub		
m18	6053	1851	A	T		TCATTGTTaATTGTAGA	TCATTGTTtATTGTAGA	sub		
m27(N)	425		A, G	-,A		AGGATTGTaTgCTATCCTG	AGGATTGT-TaCTATCCTG	compl		1
m27	2944	2519	A	T		GTTCATTAaATCGTCTG	GTTCATTAtATCGTCTG	sub	4	
m27	3558	614	A	-	-1	TTGCAGTGaCATTTTCG	TTGCAGTG-CATTTTCG	indel		
m27	4503	945	A	T		TCTCACCTaTTTCGCCA	TCTCACCTtTTTCGCCA	sub		
m27	6043	1540	A	C		CTTGTTCAaCTCATTGT	CTTGTTCAcCTCATTGT	sub		
m88(N)	600		A	G		AAATCGGAaAGAGATCC	AAATCGGAgAGAGATCC			1
m88	2479	1879	A	G		GTGTAGCAaAGGATACA	GTGTAGCAgAGGATACA	sub	5	
m88	3392	913	A	C		CCTAACAAaTCTGCAAG	CCTAACAAcTCTGCAAG	sub		
m88	4195	803	G	A		TACCAATGgATGTTGGG	TACCAATGaATGTTGGG	sub		
m88	4393	198	C	-	-1	AAATAATTcCAGTGATC	AAATAATT-CAGTGATC	indel		
m88	5238	845	A	-	-1	AGCGATGGaATTGATTC	GAATCAAT-CCATCGCT	indel		
m15	3433		A	-	-1	ACACCGGTaACAAACAC	ACACCGGT-ACAAACAC	indel	6	0
m15	3744	311	G	A		CGAAAAACgAATCATCT	CGAAAAACaAATCATCT	sub		
m15	5553	1809	G	C		AAGTGAAAgAACGGGAC	AAGTGAAAcAACGGGAC	sub		
m15	6130	577	G	A		AATATAAAgAACAATAT	AATATAAAaAACAATAT	sub		
m15	6312	182	A	G		ACACTGAAaGAGTTGGA	ACACTGAAgGAGTTGGA	sub		
m15	6330	18	T	A		TATCCAACtTCTCTATC	TATCCAACaTCTCTATC	sub		

[1] (N) - mutations in these lines were found in *NPR2* ORF; all other lines show mutations located in *LYS2* ORF

[2] Number of *npr2* mutations found in the variants, in which *NPR2* ORF has been sequenced

See also footnotes to Tables S4 and S11
