# Supplementary material for: Hypermutability of Damaged Single-Strand DNA Formed at Double-Strand Breaks and Uncapped Telomeres in Yeast Saccharomyces cerevisiae
Source: PLoS Genet. 2008 Nov 21;4(11):e1000264. doi: 10.1371/journal.pgen.1000264 (PMC2577886; doi:10.1371/journal.pgen.1000264)
Supplement: Table S13 — Separation between mutations within double and triple mutant can1 alleles. (0.03 MB PDF) [file pgen.1000264.s013.pdf]

Table S13. Separation between mutations within double and triple mutant *can1* alleles

Range of distances (nt)	Occurrence of separations within doubles (fraction)	Expected random distribution of separations within doubles (fraction)	Occurrence of separations within triples (fraction)	Expected random distribution of separations within triples (fraction)
11-148	6 (0.22)	1380 (0.14)	3 (0.20)	4175 (0.22)
149-296	1 (0.04)	1468 (0.15)	3 (0.20)	3882 (0.20)
295-444	5 (0.19)	1350 (0.14)	5 (0.33)	3110 (0.16)
445-593	4 (0.15)	1192 (0.12)	2 (0.13)	2550 (0.13)
594-737	6 (0.22)	1014 (0.10)	1 (0.07)	1930 (0.10)
738-888	2 (0.07)	878 (0.09)	1 (0.07)	1555 (0.08)
889-1184	2 (0.07)	1420 (0.14)	0	1717 (0.09)
1185-1480	1 (0.04)	886 (0.09)	0	38 (0.002)
1481-1773	0	287 (0.03)	0	94 (0.005)

Distribution of distances within multiple mutant alleles was calculated similar to (Drake et al., 2005). To estimate if UV-induced mutations were independently positioned within double and triple *can1* (Tables S6-S8 and Table S10) mutant alleles, the expected distribution of separations between random mutation events was estimated. For that purpose 10,000 random simulation trials generating multiple mutant alleles were performed for double and triple mutants separately. The trials generated 10,000 distances for double mutants and 20,000 distances for triple mutants. Since mutations separated by $\leq$ 10 nt were considered as a single event (complex mutations) those distances were excluded from calculations of expected distributions. Because of the limited numbers of mutants distances obtained from simulations were grouped into nine intervals.

Distributions of random mutations were compared with experimental results using a χ^2 test: $P\chi^2 = 0.23$ for double mutants; $P\chi^2 = 0.76$ for triple mutants

REFERENCE

Drake, J.W., Bebenek, A., Kissling, G.E., and Peddada, S. (2005). Clusters of mutations from transient hypermutability. Proc. Natl. Acad. Sci. U S A *102*, 12849-12854.
