# Supplementary material for: Hypermutability of Damaged Single-Strand DNA Formed at Double-Strand Breaks and Uncapped Telomeres in Yeast Saccharomyces cerevisiae
Source: PLoS Genet. 2008 Nov 21;4(11):e1000264. doi: 10.1371/journal.pgen.1000264 (PMC2577886; doi:10.1371/journal.pgen.1000264)
Supplement: Table S14 — Separation between mutations within double and triple mutant lys2 alleles. (0.03 MB PDF) [file pgen.1000264.s014.pdf]

Table S14. Separation between mutations within double and triple mutant *lys2* alleles

Range of distances	Occurrence of separations within doubles (fraction)	Random distribution of separations within doubles, fraction of events	Occurrence of separations within triples (fraction)	Random distribution of separations within triples, fraction of events
11-1472	9 (0.56)	5791 (0.58)	8 (0.8)	14368 (0.72)
1473-2934	6 (0.38)	3251 (0.33)	1 (0.1)	4946 (0.25)
2935-4396	1 (0.06)	921 (0.09)	1 (0.1)	528 (0.03)

Expected distributions of separations between random *lys2* mutations (Table S12) within double and triple mutant alleles were calculated similar to *can1* (see footnotes to Table S13). Because of the limited number of mutants the *lys2* data were analyzed only for three intervals of distances. Distributions within mutant alleles with more than three mutations were not calculated due to insufficient data.

Distributions of random mutations were compared with experimental results using a χ^2 test: $P\chi^2 = 0.87$ for double mutants; $P\chi^2 = 0.22$ for triple mutants.
