# Supplementary material for: Hypermutability of Damaged Single-Strand DNA Formed at Double-Strand Breaks and Uncapped Telomeres in Yeast Saccharomyces cerevisiae
Source: PLoS Genet. 2008 Nov 21;4(11):e1000264. doi: 10.1371/journal.pgen.1000264 (PMC2577886; doi:10.1371/journal.pgen.1000264)
Supplement: Table S15 — Calculated densities of UV-induced mutations. (0.02 MB PDF) [file pgen.1000264.s015.pdf]

Table S15. Calculated densities of UV-induced mutations

Target	Origin of mutants	Distribution	Number of mutations in a mutant						*X* (mutations per reporter)[3]	*D* (mutations per kb)[4]	(P_{mut})[5]	P(χ^2)[6]
			1	2	3	4	5	6				
CAN1	DSB-cen, UV (20)[1]	Observed	17	6	2	0	0	0	-	-		-
		Expected (1-3)[2]	16.3	6.5	1.7	-	-	-	0.8	0.45	0.55	0.9
		Expected (2,3)	-	5.7	1.8	-	-	-	0.95	0.53	0.61	0.8
	DSB-cen, UV (45)	Observed	21	7	4	0	0	0	-	-		-
		Expected (1-3)	12.2	10.3	5.9	-	-	-	0.85	0.48	0.57	0.6
		Expected (2,3)	-	6.5	3.0	-	-	-	1.4	0.79	0.75	0.6
subtel. *LYS2*	37°C (G2-arrest) UV (45)	Observed	7	16	5	4	1	1	-	-		-
		Expected (1-6)	9.6	10	7.4	4	1.7	0.6	2.2	0.49	0.88	0.4
		Expected (2-6)	-	15.3	7.7	2.9	0.9	0.2	1.5	0.34	0.78	0.7

Footnotes to Table S15.

[1] UV doze, J/m^2 is shown in parentheses.

[2] Mutant alleles with the multiplicity shown in parentheses were included into calculations.

[3] X - expected average number of mutations per target.

[4] D - expected average number of mutations per kilobase in *CAN1* (1773 nt) or *LYS2* (4396 nt) regions.

[5] P_{mut} - probability that the cell contains at least one mutation in a target, $(1-P_0)$, where $P_0=e^{-X}$;

[6] $P(\chi^2)$ – probability of differences between observed and expected ratios based on χ^2 distribution.
