# Supplementary material for: Hypermutability of Damaged Single-Strand DNA Formed at Double-Strand Breaks and Uncapped Telomeres in Yeast Saccharomyces cerevisiae
Source: PLoS Genet. 2008 Nov 21;4(11):e1000264. doi: 10.1371/journal.pgen.1000264 (PMC2577886; doi:10.1371/journal.pgen.1000264)
Supplement: Table S16 — Simple base sustitutions. (0.03 MB PDF) [file pgen.1000264.s016.pdf]

Table S16. Simple base sustitutions[1].

WT base (in unresected strand) [2]	Mutant base	*can1* no-DSB; UV (45)[4] (noncoding)[5]	*can1* DSB-*cen*; no UV	*can1* DSB-*cen*; UV (20)	*can1* DSB-*cen*; UV (45)	*can1* (*can1 ura3*) DSB-*cen*; UV (45)	*ura3* (*can1 ura3*) DSB-*cen*; UV (45)	*can1* DSB-*tel*; UV (45)	subtel. *LYS2* 23°C (no arrest) UV (45) (coding)[5]	subtel. *LYS2* 37°C (arrest) UV (45)	Correspon-ding mutation in dsDNA
A	G	2							1	0	AT to GC
A	T	2		1	1	1		1	3	6	AT to TA
A	C		1								AT to CG
**Total A**		4	1	1	1	1	0	1	4	6	
G	A	10	2	1					2		GC to AT
G	C	2	0								GC to CG
G	T		2	1	1			1	1		GC to TA
**Total G**		12	4	2	1	0	0	1	3	0	
**PuPu[3] context**		14	3	2	1	0	0	2	7	5	
**Total Pu**		**16**	**5**	**3**	**2**	**1**	**0**	**2**	**7**	**6**	
T	C	2	1	3	1	4	6	7		9	AT to GC
T	A	1		7	3	8	9	6	6	17	AT to TA
T	G			1		1	1	1		8	AT to CG
**Total T**		3	1	11	4	13	16	14	6	34	
C	T	5	9	5	4	8	4	7	5	14	GC to AT
C	G	1	6							2	GC to CG
C	A			1	2	3	2	2		1	GC to TA
**Total C**		6	15	6	6	11	6	9	5	17	
**PyPy-context**		8	15	15	8	22	18	20	9	45	
**Total Py**		**9**	**16**	**17**	**10**	**24**	**22**	**23**	**11**	**51**	
**Total substitu-tions**		25	21	20	12	25	22	25	18	57	

Footnotes to Table S16

[1]Complete information on sequences of mutant alleles within each category is provided in Tables S4-S12.
[2] "Unresected strand" is the strand that would remain intact after 5'-->3' resection at the end of a DSB or at uncapped telomere.
(see footnotes to Tables S5-S12 for strand assignment)
Mutations in "no-DSB" or "23oC (no arrest)" controls are assigned to the strand that would remain unresected
in the corresponding ssDNA-generating variant.
[3]Pu - purine; Py - Pyrimidine; PuPu and PyPy - two adjacent purines or pyrimidines, respectively.
(Note: PuPu in one strand would correspond to PyPy sequence in the complementary strand for mutations caused by damage in ds DNA.)
[4] UV dose, J/m^2.
[5] Noncoding strand of *CAN1* in the "no-DSB" and coding strand of *LYS2* in the "subtelomeric *LYS2*, 23°C" experiments
correspond to unresected strand in the "DSB-cen" and "subtelomeric *LYS2*, 37°C" experiments, respectively.
