# Supplementary material for: Hypermutability of Damaged Single-Strand DNA Formed at Double-Strand Breaks and Uncapped Telomeres in Yeast Saccharomyces cerevisiae
Source: PLoS Genet. 2008 Nov 21;4(11):e1000264. doi: 10.1371/journal.pgen.1000264 (PMC2577886; doi:10.1371/journal.pgen.1000264)
Supplement: Table S17 — Simple indels. (0.03 MB PDF) [file pgen.1000264.s017.pdf]

Table S17. Simple indels.

WT base (in unresected strand) [1]	*can1* no-DSB; UV (45) (noncoding)	*can1* DSB-*cen*; no UV	*can1* DSB-*cen*; UV (20)	*can1* DSB-*cen*; UV (45)	*can1* (*can1 ura3*) DSB-*cen*; UV (45)	*ura3* (*can1 ura3*) DSB-*cen*; UV (45)	*can1* DSB-*tel*; UV (45)	subtel. *LYS2* 23°C (no arrest) UV (45) (coding)	subtel. *LYS2* 37°C (arrest) UV (45)
run of A	2	3	2						
run of G		1			1				
run of T			2	2	2	6	2	2	4
run of C		1	1			2			
**Total indels[2]**	**9**	**6**	**9**	**3**	**4**	**12**	**2**	**2**	**19**

[1]Only indel mutations in which deleted or inserted base(s) can be unambiguously assigned to a homonucleotide run were categorized

[2] Includes all simple indel mutations, regardless of the presence of a homonucleotide run.

Also see footnotes to Table S16.
