# Supplementary material for: Hypermutability of Damaged Single-Strand DNA Formed at Double-Strand Breaks and Uncapped Telomeres in Yeast Saccharomyces cerevisiae
Source: PLoS Genet. 2008 Nov 21;4(11):e1000264. doi: 10.1371/journal.pgen.1000264 (PMC2577886; doi:10.1371/journal.pgen.1000264)
Supplement: Table S18 — Base substitutions within complex mutations. (0.03 MB PDF) [file pgen.1000264.s018.pdf]

Table S18. Base substitutions within complex mutations.

WT base (in unresected strand)	*can1* no-DSB; UV (45) (noncoding)	*can1* DSB-*cen*; no UV	*can1* DSB-*cen*; UV (20)	*can1* DSB-*cen*; UV (45)	*can1* (*can1 ura3*) DSB-*cen*; UV (45)	*ura3* (*can1 ura3*) DSB-*cen*; UV (45)	*can1* DSB-*tel*; UV (45)	subtel. *LYS2* 23°C (no arrest) UV (45) (coding)	subtel. *LYS2* 37°C (arrest) UV (45)
Pu	7					1			1
Py	12	2	10	6	3	6	2		6
**Total complex mutations**[1]	11	1	6	3	2	3	1	0	5

[1] Includes all complex mutations regardless of the number and types of changes within a complex mutation.

Also see footnotes to Table S16.
