# Supplementary material for: Hypermutability of Damaged Single-Strand DNA Formed at Double-Strand Breaks and Uncapped Telomeres in Yeast Saccharomyces cerevisiae
Source: PLoS Genet. 2008 Nov 21;4(11):e1000264. doi: 10.1371/journal.pgen.1000264 (PMC2577886; doi:10.1371/journal.pgen.1000264)
Supplement: Text S1 — Supplemental calculations. (0.02 MB PDF) [file pgen.1000264.s019.pdf]

Text S1 - Supplemental Calculations

SUPPLEMENTAL CALCULATIONS

Utilizing iterations to solve a truncated Poisson distribution, similar to that of (Drake et al., 2005), we estimated the density of UV-induced mutations in *CAN1* and *LYS2* based only on alleles with multiple mutations as well as based on the entire experimental distributions (Table 1, Table S15). First the probability P(χ^2) was determined by χ^2- test comparing distribution observed in a group and the expected Poisson distribution with average number of mutations in a target (*CAN1* or *LYS2*) X=1. In the expected Poisson distribution, only the members with the numbers of mutations present in the experimental distribution were calculated. Then the calculation of P(χ^2) was repeated for X=2. The size and direction of each next step was chosen with the goal of increasing P(χ^2) based on comparison of the two previous steps. The iterations were stopped when the steps were as little as 0.05. (Note: the distribution of multiples within the "DSB-*tel*, UV" group could not be approximated by Poisson formula with likelihood >0.05 (not shown).)

RFEFERENCE

Drake, J.W., Bebenek, A., Kissling, G.E., and Peddada, S. (2005). Clusters of mutations from transient hypermutability. Proc. Natl. Acad. Sci. U S A *102*, 12849-12854.
